# MRI-Derived Muscle Composition and Visceral Adiposity in Relation to Mortality and Vascular Outcomes in MASLD

**DOI:** 10.3390/diagnostics16213487

**Published:** 2026-10-24

**Authors:** Tom Ryu, Beom Sun Chung, Yoon Kang, Jaejun Lee, Ji Won Han, Hyun Yang, Keungmo Yang

**Affiliations:** 1 Department of Internal Medicine, Institute for Digestive Research, Digestive Disease Center, Soonchunhyang University College of Medicine, Seoul 04401, Republic of Korea; tomryu1@schmc.ac.kr; 2 Department of Anatomy, Yonsei University Wonju College of Medicine, Wonju 26426, Republic of Korea; bschung@yonsei.ac.kr; 3 Division of Gastroenterology and Hepatology, Department of Internal Medicine, College of Medicine, The Catholic University of Korea, Seoul 06591, Republic of Korea; whitia12@catholic.ac.kr (Y.K.); pwln0516@gmail.com (J.L.); tmznjf@catholic.ac.kr (J.W.H.); oneggu@naver.com (H.Y.)

**Keywords:** metabolic dysfunction-associated steatotic liver disease, magnetic resonance imaging, muscle composition, prognosis

## Abstract

**Background/Objectives**: Metabolic dysfunction-associated steatotic liver disease (MASLD) is a cardiometabolic phenotype rather than a single-pathway liver disorder, yet prognostic work tends to privilege liver fat over the rest of body composition. We examined which magnetic resonance imaging (MRI)-derived compartment, including hepatic, abdominal adipose, or skeletal muscle, best explained risk within MASLD. **Methods**: We investigated 24,976 UK Biobank adults with abdominal MRI, including 10,490 with MASLD, 2577 with MASLD with increased alcohol intake, and 11,909 without steatotic liver disease. Five MRI parameters were examined: proton density fat fraction (PDFF), visceral adipose tissue (VAT), abdominal subcutaneous adipose tissue (ASAT), muscle fat infiltration (MFI), and thigh fat-free muscle volume (FFMV). Primary outcomes were all-cause mortality, incident cardiovascular disease, and incident cerebrovascular disease. Cox models jointly included all five MRI variables and adjusted for demographic, metabolic, and biochemical covariates. **Results**: Within MASLD, liver PDFF was not independently associated with all-cause mortality or cerebrovascular disease after multivariable adjustment, whereas an inverse association was observed with cardiovascular disease. By contrast, higher MFI was associated with all-cause mortality and cerebrovascular disease, whereas greater FFMV was associated with lower cardiovascular risk. VAT showed an independent association with cardiovascular disease, whereas ASAT had no significance. Clustering identified a lean-sarcopenic phenotype with higher mortality and cerebrovascular risk than a balanced reference phenotype. **Conclusions**: MRI-derived body-composition parameters showed outcome-specific associations with clinical outcomes in MASLD. Muscle-related measures were particularly associated with all-cause mortality and cerebrovascular disease, whereas visceral adiposity and muscle volume contributed to cardiovascular risk. These findings support the potential value of integrated body-composition assessment rather than reliance on hepatic steatosis alone, although further validation is needed to establish its incremental clinical utility.

## 1. Introduction

The Metabolic dysfunction-associated steatotic liver disease (MASLD) is the most common chronic liver disease globally, affecting approximately 38% of adults and projected to exceed 50% by 2040 [1]. MASLD is defined by the presence of hepatic steatosis together with at least one cardiometabolic risk factor, including overweight or obesity, impaired glucose metabolism, hypertension, hypertriglyceridemia, or low high-density lipoprotein (HDL) cholesterol. Within the steatotic liver disease spectrum, MASLD with increased alcohol intake (MetALD) is defined in individuals with MASLD who consume alcohol above conventional thresholds (140–350 g/week in women and 210–420 g/week in men), representing an overlap between metabolic and alcohol-related liver injury. This updated framework demonstrates a shift from exclusion-based definitions from non-alcoholic fatty liver disease, toward a positive, pathophysiology-driven classification of steatotic liver disease [2].

MASLD has reframed fatty liver disease as part of a wider metabolic disorder rather than a liver-limited condition [3]. It reflects the reality that the burden carried by patients with steatotic liver disease is not determined by hepatic triglyceride accumulation alone, but by the combined effects of visceral adiposity, insulin resistance, dyslipidemia, vascular risk, and changes in skeletal muscle [4,5,6]. This is precisely why the clinical course of MASLD is heterogeneous. Some individuals remain metabolically stable for years, whereas others develop cardiovascular and cerebrovascular disease or die prematurely without progressing to overt end-stage liver disease. Therefore, the practical question is not only whether hepatic steatosis is present, but also which physiological compartments explain why one adult with MASLD does poorly while another does not [7].

Most prognostic studies approach this question one compartment at a time. Liver-focused work presents the amount of steatosis, fibrosis stage, or transaminase activity. Cardiometabolic studies often emphasize abdominal adiposity, particularly visceral fat, whereas sarcopenia-oriented research focuses on muscle mass or muscle attenuation [8,9,10]. Because MASLD lies at the intersection of these processes, assessing one compartment in isolation risks confounding shared prognostic signals. Magnetic resonance imaging (MRI) is particularly attractive in this setting because it can quantify multiple relevant compartments in the same individual, non-invasively and at scale [11]. UK Biobank has shown that multi-compartment body-composition phenotyping is feasible in large populations and that fat depots carry distinct metabolic implications [12,13]. What remains uncertain is how these compartments behave among individuals who already meet MASLD criteria.

There are several reasons to suspect that liver fat alone may provide an incomplete assessment of prognosis within MASLD. MRI-derived proton density fat fraction (PDFF) is an established quantitative imaging biomarker of hepatic fat content [14]. However, hepatic steatosis alone may not adequately capture systemic cardiometabolic risk or physiological vulnerability. Previous studies have suggested that adverse muscle composition, characterized by greater muscle fat infiltration and lower muscle volume, is associated with impaired physical function, metabolic comorbidities, and increased mortality [15].

Therefore, we used the UK Biobank abdominal MRI cohort to evaluate five MRI-derived body-composition parameters (PDFF, visceral adipose tissue [VAT], abdominal subcutaneous adipose tissue [ASAT], muscle fat infiltration [MFI], and fat-free muscle volume [FFMV]) in relation to all-cause mortality, incident cardiovascular disease, and incident cerebrovascular disease. We quantified independent contribution of each MRI-derived parameter, evaluated dose–response relationships, assessed subgroup consistency, compared model discrimination, and translated key signals into unsupervised MASLD phenotypes.

## 2. Materials and Methods

### 2.1. Study Design and Source Population

UK Biobank is a prospective population-based resource that enrolled more than 500,000 participants in the United Kingdom and subsequently recalled a large subset for imaging [16,17]. The present analysis was based on participants who had available abdominal MRI data and for whom the completed analytic dataset contained the MRI body-composition variables and baseline clinical information required for multivariable modelling (Category 149, abdominal MRI and abdominal composition). All participants provided written informed consent, and the present work used de-identified data under an approved UK Biobank application. The UK Biobank Application ID for this research is 117214, with ethical approval obtained from the North West Multi-Centre Research Ethics Committee.

Participants were excluded sequentially for unavailable abdominal MRI, incomplete clinical data, autoimmune hepatitis, primary biliary cholangitis, chronic hepatitis B, chronic hepatitis C, cryptogenic steatotic liver disease, alcohol-associated liver disease, prior malignancy, and prior hepatic decompensation and cardio-cerebrovascular disease. After these exclusions, 24,976 adults remained for the principal analysis. They were classified into three mutually exclusive groups, No steatotic liver disease (No SLD), MASLD, and MASLD with increased alcohol intake (MetALD), according to the prespecified study definitions. MASLD was defined by the presence of hepatic steatosis together with at least one cardiometabolic risk factor (body mass index [BMI] ≥ 25 kg/m^2^ [≥23 kg/m^2^ in Asians] or increased waist circumference; fasting glucose ≥ 100 mg/dL, HbA1c ≥ 5.7%, or type 2 diabetes; blood pressure ≥ 130/85 mmHg or antihypertensive treatment; triglycerides ≥ 150 mg/dL or lipid-lowering therapy; or HDL cholesterol ≤ 40 mg/dL in men or ≤50 mg/dL in women), while MetALD was defined in individuals meeting MASLD criteria who reported alcohol consumption of 140–350 g/week in women or 210–420 g/week in men (Supplementary Figure S1) [2]. Hepatic steatosis was operationally defined using the fatty liver index (FLI), with a threshold of ≥60. The FLI was calculated from body mass index, waist circumference, triglyceride levels, and gamma-glutamyl transferase. MASLD was defined as FLI ≥ 60 together with at least one cardiometabolic risk factor, while MetALD was defined according to the corresponding alcohol consumption thresholds. MRI-derived PDFF was evaluated separately as a continuous imaging-derived marker of hepatic fat content and was not used to define the study population. The available follow-up variables were derived from the initial UK Biobank recruitment assessment rather than the subsequent MRI examination. Accordingly, follow-up duration was calculated from the initial assessment, whereas MRI-derived body-composition parameters were obtained during subsequent imaging visits. Demographic and clinical covariates were obtained from the available assessment records. This temporal distinction was considered when interpreting the associations between MRI-derived parameters and clinical outcomes.

### 2.2. MRI Body-Composition Variables

Abdominal MRI data in the UK Biobank were acquired using a standardized protocol on a 1.5-T Siemens MAGNETOM Aera scanner (Siemens Healthineers, Erlangen, Germany). A dual-echo Dixon VIBE sequence provided water- and fat-separated volumetric images extending from the neck to the knees, while a dedicated multi-echo acquisition was used for liver PDFF assessment. MRI-derived body-composition measurements were obtained through standardized image-processing procedures involving image calibration, image-stack fusion, tissue segmentation, and quantitative assessment of adipose and skeletal-muscle compartments. Liver PDFF was derived from predefined hepatic regions of interest, avoiding major vessels and bile ducts. Quality-control procedures were incorporated into the imaging-processing workflow, as described in previous UK Biobank methodological studies [12].

Five continuous MRI-derived variables were selected initially because they represent the hepatic, abdominal adipose, and skeletal-muscle compartments most relevant to the central study question. PDFF (%) was used as the quantitative marker of hepatic steatosis. VAT (L) and ASAT (L) represented abdominal visceral and subcutaneous adipose tissue compartments. MFI (%) captured myosteatosis, and total thigh FFMV (L) represented lower-limb muscle quantity [12,18,19]. Additional descriptive body-composition measures included the weight-to-muscle ratio, calculated as body weight divided by total thigh FFMV; total trunk fat volume, representing the quantified adipose tissue volume in the trunk; and total abdominal adipose tissue index, representing abdominal adipose tissue volume normalized to height squared.

All measurements were obtained at the imaging visit using the UK Biobank MRI acquisition and image-processing pipeline. The main analyses used the MRI variables on their original scales to preserve direct interpretability. Hazard ratios were therefore expressed per 1% higher PDFF, per 1 L higher VAT or ASAT, per 1% higher MFI, and per 1 L higher FFMV. Standardized values were used in the clustering analyses, where comparability across scales was required. The MRI-derived parameters were analyzed on their original measurement scales to preserve interpretability within each imaging compartment. Because the measurement units differed across parameters, the resulting hazard ratios were not intended for direct comparisons of effect magnitude between compartments.

The MFI variable used in the primary analyses represented overall thigh muscle fat infiltration, whereas regional anterior and posterior thigh measurements were additionally presented for descriptive purposes. Total thigh FFMV was analyzed as an absolute volume, with sex and BMI included as adjustment variables in the multivariable models.

### 2.3. Outcomes

The three outcomes of interest were all-cause mortality, incident cardiovascular disease, and incident cerebrovascular disease. Mortality was ascertained through national death registries. Cardiovascular and cerebrovascular events were identified through linked hospital and registry data using the definitions prespecified in the completed study dataset [16,20]. Cardiovascular and cerebrovascular outcomes were identified from linked UK Biobank hospital and registry records using predefined International Classification of Diseases, 10th Revision (ICD-10) codes I20–I25 and I60–I69, respectively. Cardiovascular and cerebrovascular outcomes were identified using the available linked health records. Follow-up intervals were based on the initial UK Biobank assessment rather than the subsequent MRI examination. Accordingly, the temporal relationship between MRI acquisition and recorded clinical events could not be fully established.

### 2.4. Covariates

The adjustment strategy used three nested models. Model 1 included age and sex. Model 2 added BMI, diabetes, dyslipidemia, hypertension, and smoking. Model 3 added alanine aminotransferase, aspartate aminotransferase, gamma-glutamyl transferase, total bilirubin, albumin, and platelet count. This staging was chosen to answer two related questions: whether the MRI markers retained prognostic information after conventional demographic adjustment, and whether any such information survived further control for the metabolic and hepatic environment in which MASLD is usually interpreted.

### 2.5. Statistical Analysis

The primary time-to-event analyses used Cox proportional-hazards regression. Models were fitted in the total cohort and separately within each SLD classification. To assess dose–response shape, restricted cubic splines with four knots at the 5th, 35th, 65th, and 95th percentiles were fitted for each MRI parameter, while the remaining MRI markers were retained as linear terms and the full Model 3 covariate set was preserved. Hazard ratios (HRs) were plotted across the 2nd to 98th percentiles, using the median as the reference. For cardiovascular and cerebrovascular outcomes, Fine–Gray subdistribution hazard models were additionally fitted as sensitivity analyses, treating death before the respective event as a competing event. The same covariate adjustment strategy as Model 3 was applied. Subgroup and interaction analyses were considered exploratory because of the number of comparisons performed, with interpretation emphasizing effect estimates and 95% confidence intervals.

Subgroup analyses were performed across categories of sex, age, obesity, diabetes, hypertension, and dyslipidemia. Incremental discrimination was assessed using MRI-only nested models: M1, PDFF alone; M2, PDFF plus VAT and ASAT; and M3, PDFF plus MFI and FFMV. Harrell’s concordance index was calculated for each model, and pairwise differences were examined. To evaluate the potential influence of underlying liver fibrosis, a sensitivity analysis was performed by restricting the MASLD cohort to participants with FIB-4 < 1.45. FIB-4 was calculated as age (years) × AST (U/L)/[platelet count (10^9^/L) × √ALT (U/L)]. Because these components were already included in the fully adjusted model, FIB-4 was not additionally incorporated as a covariate in Model 3.

To derive clinically interpretable phenotypes, unsupervised k-means clustering was performed in the MASLD cohort using standardized PDFF, MFI, and FFMV. The optimal number of clusters was selected by the average silhouette width, and associations between clusters and outcomes were then tested using Model 3-adjusted Cox models. The clustering analysis was designed to characterize liver–muscle phenotypes using PDFF, MFI, and FFMV, representing hepatic fat accumulation, muscle quality, and muscle quantity, respectively. VAT was not included because visceral adiposity was evaluated separately in the association and discrimination analyses. To assess the potential influence of sex-related differences in body composition, a sensitivity analysis was performed in which PDFF, MFI, and FFMV were standardized separately within men and women before repeating the k-means clustering. Associations between the resulting phenotypes and clinical outcomes were evaluated using the same multivariable adjustment strategy as in the primary analysis. The term “lean-sarcopenic” was used as a descriptive label for an imaging-derived phenotype characterized by relatively lower muscle volume and higher muscle fat infiltration, rather than as a clinical diagnosis of sarcopenia or an indication of low BMI.

All tests were two-sided, and *p* < 0.05 was considered statistically significant. Analyses were performed in R version 4.5.0. (R Foundation for Statistical Computing, Vienna, Austria).

## 3. Results

### 3.1. Baseline Characteristics and Distribution of MRI Markers

The analytic sample comprised 24,976 adults, of whom 11,909 were classified as No SLD, 10,490 as MASLD, and 2577 as MetALD. Mean age was 54.52 ± 7.43 years overall, and 46.4% of participants were male (Table 1). Mean BMI was 28.86 ± 3.71 kg/m^2^ in MASLD versus 23.48 ± 2.24 kg/m^2^ in No SLD, diabetes was present in 9.4% versus 1.8%, hypertension in 42.5% versus 20.5%, and dyslipidemia in 34.4% versus 16.3%. The MetALD group was older, more predominantly male, and showed the highest prevalence of smoking, hypertension, and dyslipidemia.

The MRI compartments showed similarly clear separation across strata. Mean PDFF rose from 2.90 ± 2.44% in No SLD to 6.35 ± 5.67% in MASLD and 6.09 ± 5.32% in MetALD. Visceral adiposity was more than doubled in MASLD relative to No SLD (4.81 ± 2.09 vs. 2.10 ± 1.14 L), with similar differences for total abdominal adiposity. MFI was also higher in MASLD and MetALD than in No SLD, whereas total thigh FFMV was higher in the MASLD and MetALD classification (Table 1). The distributions of MRI-derived body composition parameters showed a consistent shift across disease classification, with separation evident across the full range of values rather than being limited to extreme values. Also, this pattern was preserved after stratification by sex (Supplementary Figure S2).

The five MRI variables were correlated but not redundant. In the MASLD cohort, PDFF and VAT showed a moderate positive correlation, while MFI and FFMV showed a moderate inverse correlation (Supplementary Figure S3).

Hepatic and abdominal fat variables (PDFF, VAT, ASAT) showed positive correlations with each other, whereas muscle-related variables (MFI and FFMV) formed a separate, internally convergent module characterized by inverse correlation between muscle quality and quantity. These findings suggest that hepatic, adipose, and muscle compartments represent distinct domains, supporting joint modelling to assess their independent contributions (Supplementary Figure S4).

### 3.2. Independent Associations of MRI Body Composition with Outcomes

**Figure 1:**
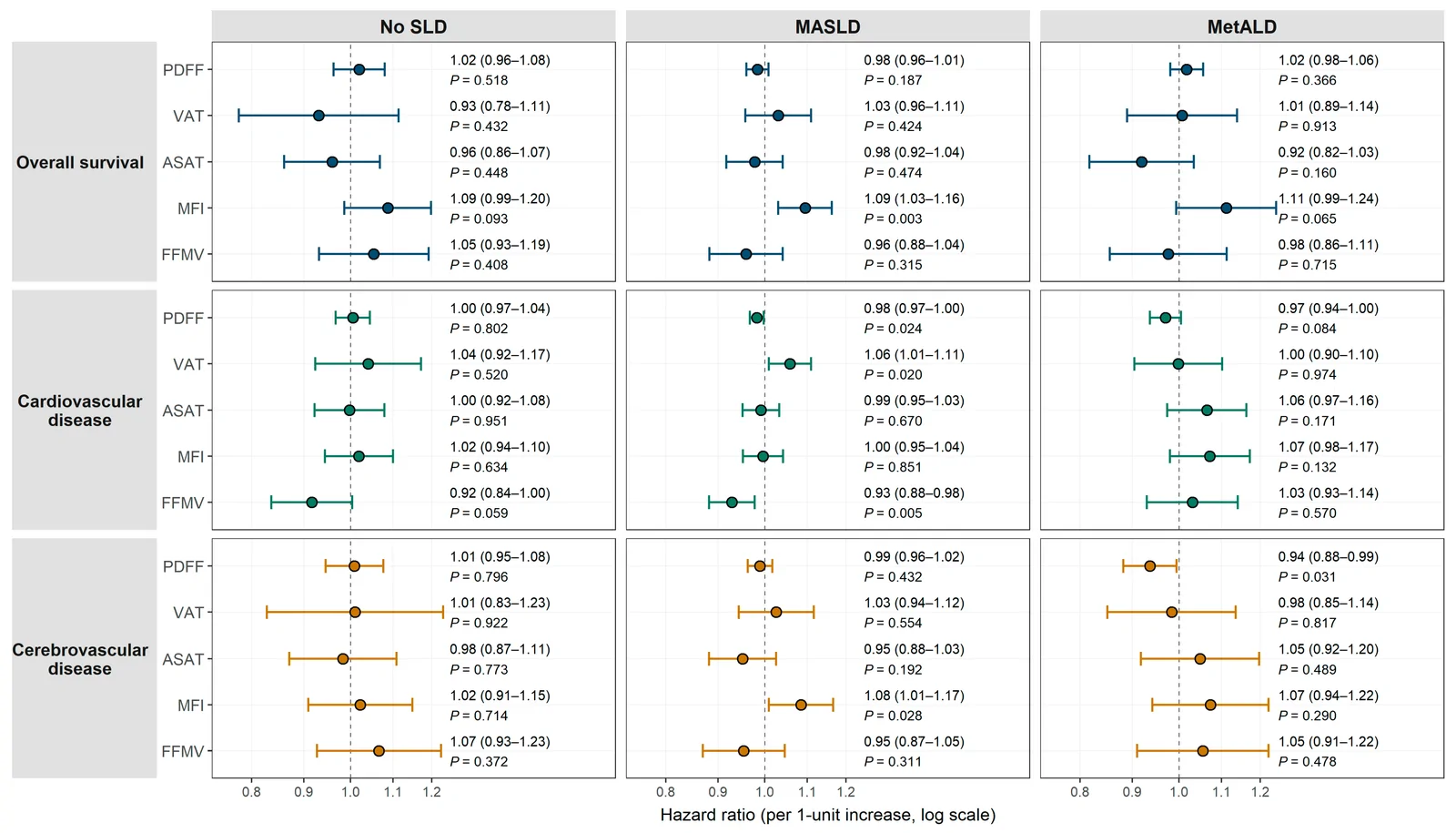
Associations of MRI body-composition parameters with clinical outcomes across SLD classification. Forest plots of fully adjusted hazard ratios for overall survival, incident cardiovascular disease, and incident cerebrovascular disease in the No SLD, MASLD, and MetALD cohorts. Dots indicate hazard ratios and bars indicate 95% confidence intervals. SLD, steatotic liver disease; MASLD, metabolic dysfunction-associated steatotic liver disease; MetALD, MASLD with increased alcohol intake; PDFF, proton density fat fraction; VAT, visceral adipose tissue; ASAT, abdominal subcutaneous adipose tissue; MFI, muscle fat infiltration; FFMV, fat-free muscle volume.

After full adjustment for demographic, metabolic, biochemical, and imaging covariates, PDFF was not associated with all-cause mortality (HR 0.98 per 1%, 95% CI 0.96–1.01; *p* = 0.187) or incident cerebrovascular disease (HR 0.99, 95% CI 0.96–1.02; *p* = 0.432) in MASLD. In contrast, muscle-related parameters showed more consistent associations. Higher MFI was associated with increased risk of all-cause mortality (HR 1.09 per 1%, 95% CI 1.03–1.16; *p* = 0.003) and cerebrovascular disease (HR 1.08, 95% CI 1.01–1.17; *p* = 0.028), while total thigh FFMV was inversely associated with incident cardiovascular disease (HR 0.93 per 1 L, 95% CI 0.88–0.98; *p* = 0.005). For cardiovascular disease, VAT also retained an independent association (HR 1.06 per 1 L, 95% CI 1.01–1.11; *p* = 0.020), whereas ASAT was not associated with any outcome after adjustment (Table 2, Figure 1).

Outside the MASLD classification, the pattern was less consistent. In the total cohort, MFI remained associated with all-cause mortality (HR 1.10, 95% CI 1.05–1.15; *p* < 0.001) and cerebrovascular disease (HR 1.07, 95% CI 1.01–1.13; *p* = 0.019), while FFMV was inversely associated with cardiovascular disease (HR 0.95, 95% CI 0.91–0.99; *p* = 0.007) after full adjustment (Supplementary Table S1, Figure 1). In participants without SLD, most associations attenuated after adjustment, with no MRI parameter showing consistent independent associations across outcomes (Supplementary Table S2, Figure 1). No consistent associations were observed across outcomes after full adjustment (Supplementary Table S3, Figure 1). Overall, associations were more consistent within the MASLD cohort than in other groups.

### 3.3. Dose–Response Relations

**Figure 2:**
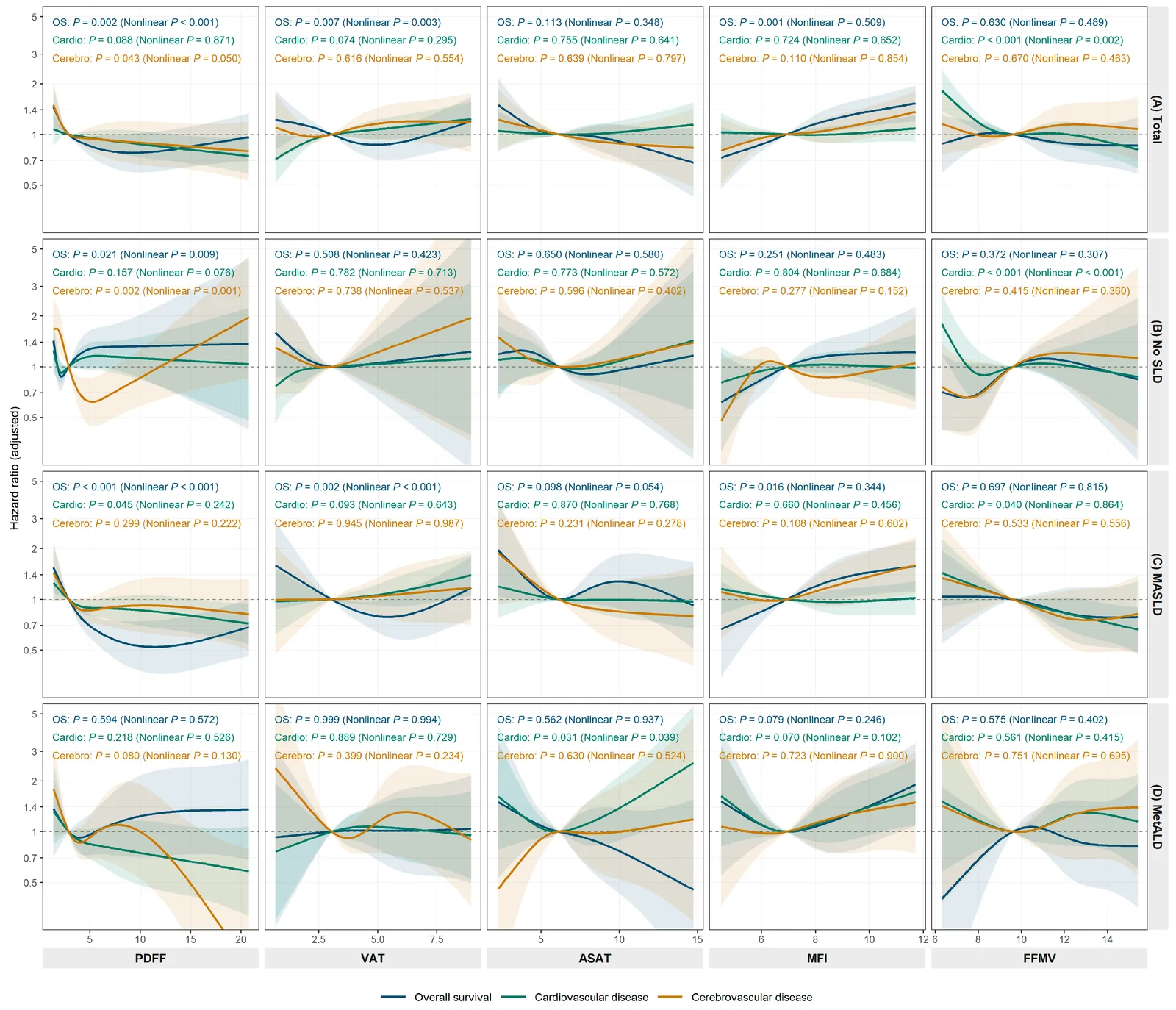
Dose–response associations of MRI body-composition parameters with clinical outcomes across SLD classification. Restricted cubic spline curves for PDFF, VAT, ASAT, MFI, and FFMV in the total cohort and in each SLD classification. Solid lines show adjusted hazard ratios for overall survival, cardiovascular disease, and cerebrovascular disease, and shaded bands indicate 95% confidence intervals. Overall and non-linearity *p* values are shown in each panel. OS, overall survival; SLD, steatotic liver disease; MASLD, metabolic dysfunction-associated steatotic liver disease; MetALD, MASLD with increased alcohol intake; PDFF, proton density fat fraction; VAT, visceral adipose tissue; ASAT, abdominal subcutaneous adipose tissue; MFI, muscle fat infiltration; FFMV, fat-free muscle volume.

Restricted cubic spline analyses were used to assess the shape of the dose–response relationships across MRI-derived compartments. In MASLD, MFI showed a positive association with all-cause mortality (*p* = 0.016), indicating a predominantly linear increase in risk across the observed range. Similarly, FFMV demonstrated an inverse association with incident cardiovascular disease (*p* = 0.040), consistent with a gradual reduction in risk at higher levels of muscle volume. PDFF showed a statistically significant association with all-cause mortality (*p* < 0.001) with evidence of non-linearity (*p* < 0.001), with spline curves demonstrating a non-monotonic pattern across the distribution. ASAT showed no significant associations across outcomes (mortality *p* = 0.098; cardiovascular *p* = 0.870; cerebrovascular *p* = 0.231), and VAT also demonstrated no significant association with cardiovascular disease (*p* = 0.093). Across the total cohort, MFI was associated with all-cause mortality (*p* < 0.001), and FFMV was associated with cardiovascular disease (*p* < 0.001), both without evidence of non-linearity. In the no SLD and MetALD cohort, spline estimates were attenuated and less consistent, with no consistent patterns observed across outcomes (Figure 2). Taken together, dose–response analyses indicate that muscle-related parameters show consistent linear associations with outcomes, whereas liver fat demonstrates non-linear and less consistent patterns.

### 3.4. Consistency of Associations Across Clinical Subgroups

Interaction analyses were performed within the MASLD cohort to evaluate potential effect modification across categories of sex, age, obesity, diabetes, hypertension, and dyslipidemia.

Associations of MFI and FFMV with clinical outcomes were generally consistent across subgroups, with most estimates remaining on the same side of the null. Higher MFI was associated with increased risk of all-cause mortality and cerebrovascular disease, whereas higher FFMV was associated with lower risk of cardiovascular disease. Interaction tests showed limited evidence of effect modification, with only a small number of nominally significant interactions, including MFI with cerebrovascular disease by sex and dyslipidemia status, and FFMV with cardiovascular disease by age.

**Figure 3:**
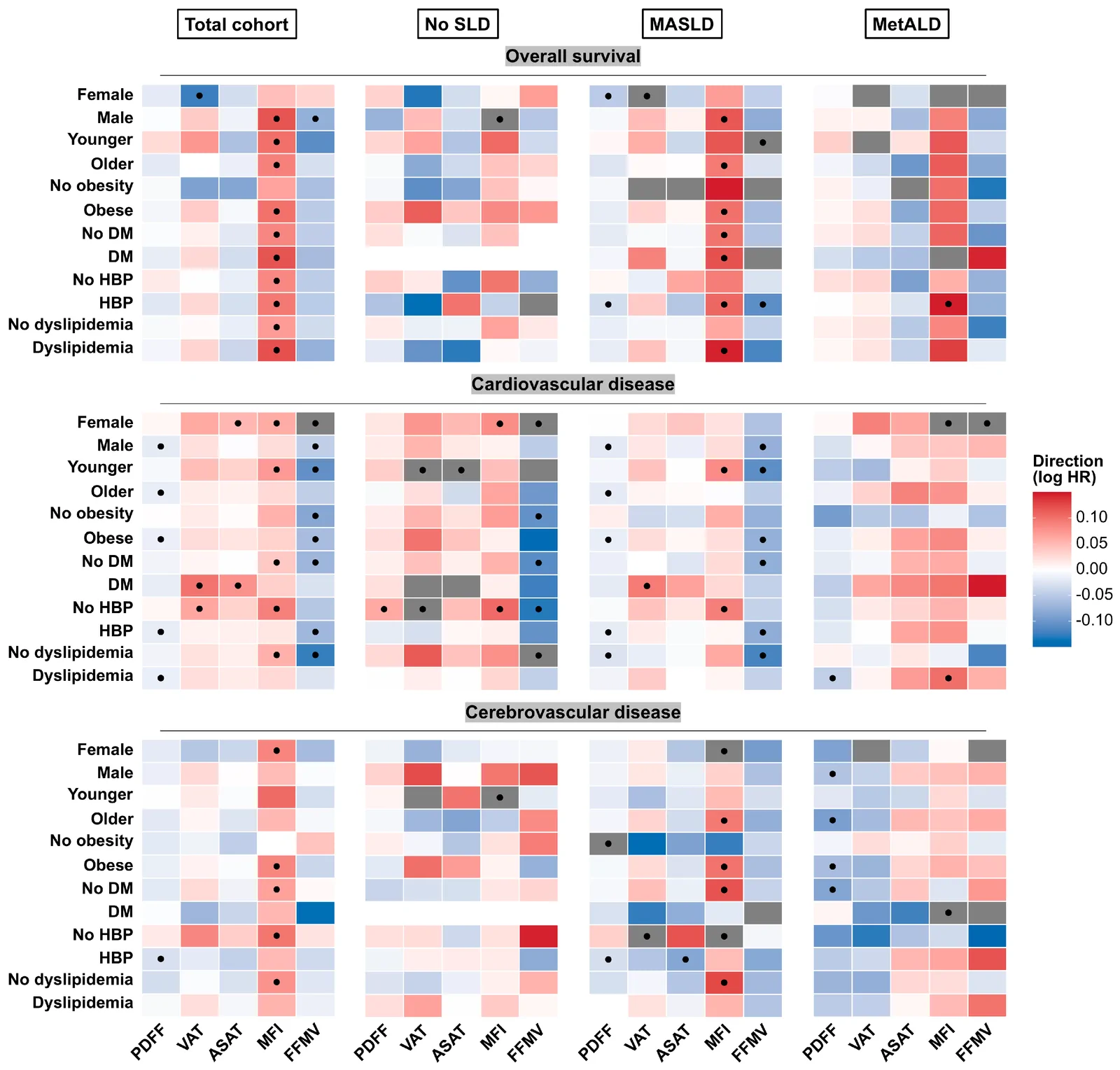
Interaction analyses of MRI–outcome associations across clinical subgroups. Heatmap of subgroup-specific log-hazard ratios for MRI-derived body composition parameters according to sex, age, obesity, diabetes, hypertension, and dyslipidemia. Red indicates higher risk and blue indicates lower risk. Dots indicate nominally significant interaction effects (*p* for interaction < 0.05), and gray cells indicate estimates not available. DM, diabetes mellitus; HBP, high blood pressure; SLD, steatotic liver disease; MASLD, metabolic dysfunction-associated steatotic liver disease; MetALD, MASLD with increased alcohol intake; PDFF, proton density fat fraction; VAT, visceral adipose tissue; ASAT, abdominal subcutaneous adipose tissue; MFI, muscle fat infiltration; FFMV, fat-free muscle volume. Age was categorized as <65 and ≥65 years, and obesity was defined as BMI ≥ 25 kg/m^2^.

In contrast, similar analyses in the No SLD and MetALD cohorts showed less consistent patterns (Figure 3).

Overall, these findings indicate that the prognostic associations of MRI-derived muscle parameters are largely consistent across clinical subgroups, and that the patterns observed in the total cohort are primarily driven by the significant associations within the MASLD cohort.

### 3.5. Incremental Discrimination of Nested MRI Models

**Figure 4:**
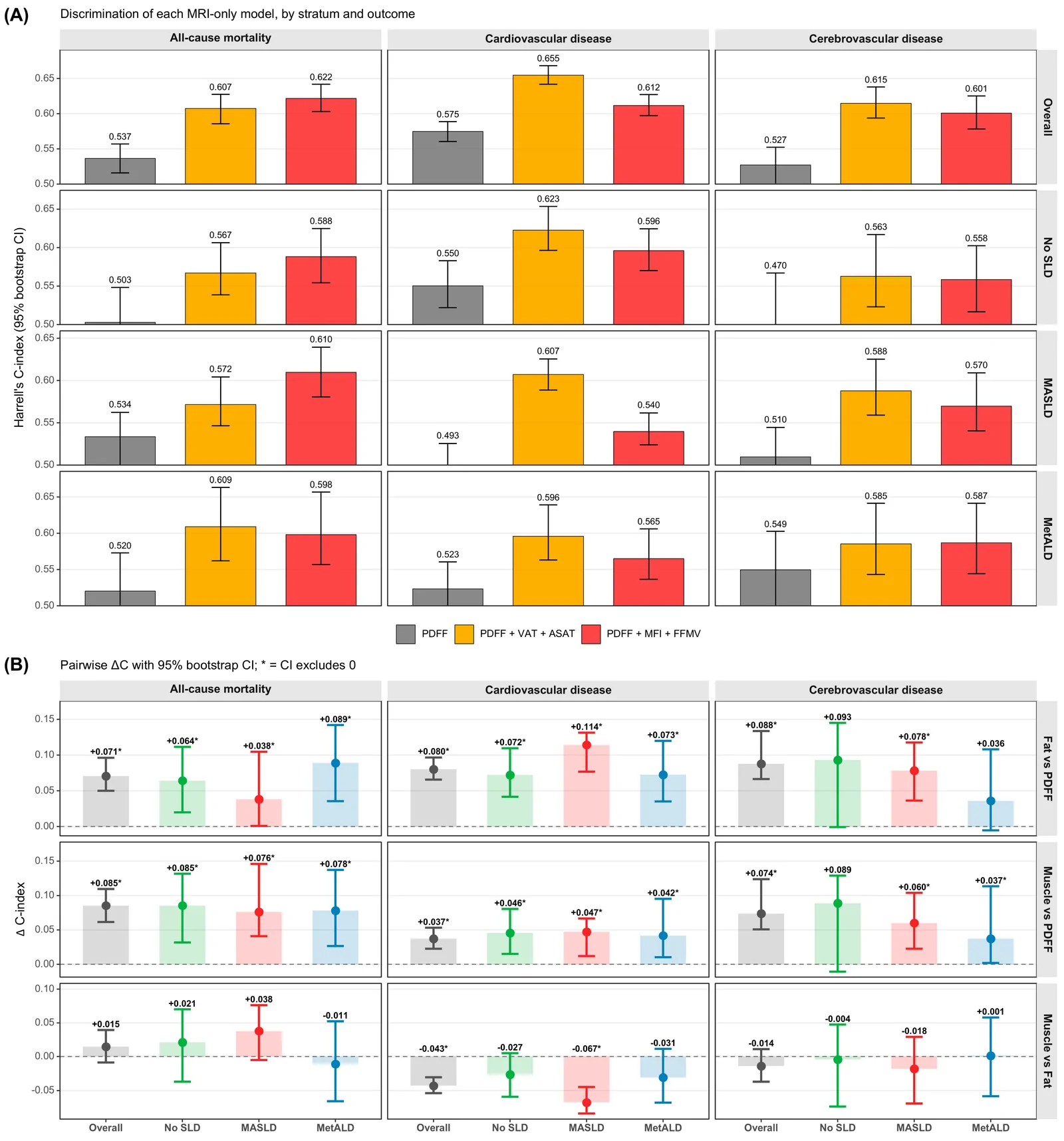
Discrimination of nested MRI-only models across SLD classification. (**A**) Harrell’s C-index for three MRI-only models: M1, PDFF alone; M2, PDFF plus VAT and ASAT; and M3, PDFF plus MFI and FFMV. (**B**) Pairwise differences in C-index for the three model comparisons. Error bars indicate 95% confidence intervals; an asterisk indicates that the confidence interval does not cross zero. CI, confidence interval; SLD, steatotic liver disease; MASLD, metabolic dysfunction-associated steatotic liver disease; MetALD, MASLD with increased alcohol intake; PDFF, proton density fat fraction; VAT, visceral adipose tissue; ASAT, abdominal subcutaneous adipose tissue; MFI, muscle fat infiltration; FFMV, fat-free muscle volume.

To evaluate whether MRI-derived parameters improved risk discrimination beyond association, three nested MRI-only models were compared: PDFF alone, PDFF plus abdominal adiposity (VAT and ASAT), and PDFF plus muscle measures (MFI and FFMV). Across cohorts, the PDFF-only model showed limited discrimination for all outcomes. In the MASLD cohort, the apparent C-index for all-cause mortality was 0.534, increasing to 0.572 with VAT and ASAT and to 0.610 with the inclusion of muscle measures (Supplementary Table S4, Figure 4). After bootstrap correction, the corresponding C-indices were 0.527, 0.566, and 0.604, respectively (Supplementary Table S5). For cardiovascular disease in MASLD, the apparent C-index increased from 0.493 (PDFF) to 0.607 (VAT + ASAT) and 0.540 (muscle), with bootstrap-corrected values of 0.487, 0.605, and 0.536, respectively. For cerebrovascular disease, the corresponding apparent C-indices were 0.510, 0.588, and 0.570, and bootstrap-corrected values were 0.501, 0.580, and 0.562 (Supplementary Tables S4 and S5, Figure 4). These findings indicate that models incorporating abdominal adiposity and muscle parameters improve risk discrimination beyond liver fat alone, supporting the prognostic relevance of non-hepatic body composition in MASLD.

In the total cohort, similar patterns were observed, but the magnitude of incremental improvement in discrimination was smaller than in the MASLD cohort. In participants without SLD, incremental improvements in discrimination were smaller than those observed in the MASLD cohort, while in the MetALD cohort, discrimination improved with extended models but differences between adiposity and muscle parameters were less pronounced (Supplementary Tables S4 and S5, Figure 4).

Bootstrap optimism was minimal across all models (generally ≤0.01), and calibration slopes for extended MRI models were close to 1.0, supporting the importance of the observed discrimination gains (Supplementary Table S5).

### 3.6. Unsupervised Phenotypes Within MASLD

Although outcome differences across SLD classifications were evident at the population level, clustering within MASLD identified subgroups with distinct prognostic profiles. Unsupervised clustering was performed within the MASLD cohort using standardized PDFF, MFI, and FFMV. The optimal number of clusters was determined using silhouette analysis, the elbow method, and cluster size distribution, all of which supported a three-cluster solution (Supplementary Figure S6), comprising balanced (n = 5239), lean-sarcopenic (n = 4001), and steatotic-dominant (n = 1250) groups.

**Figure 5:**
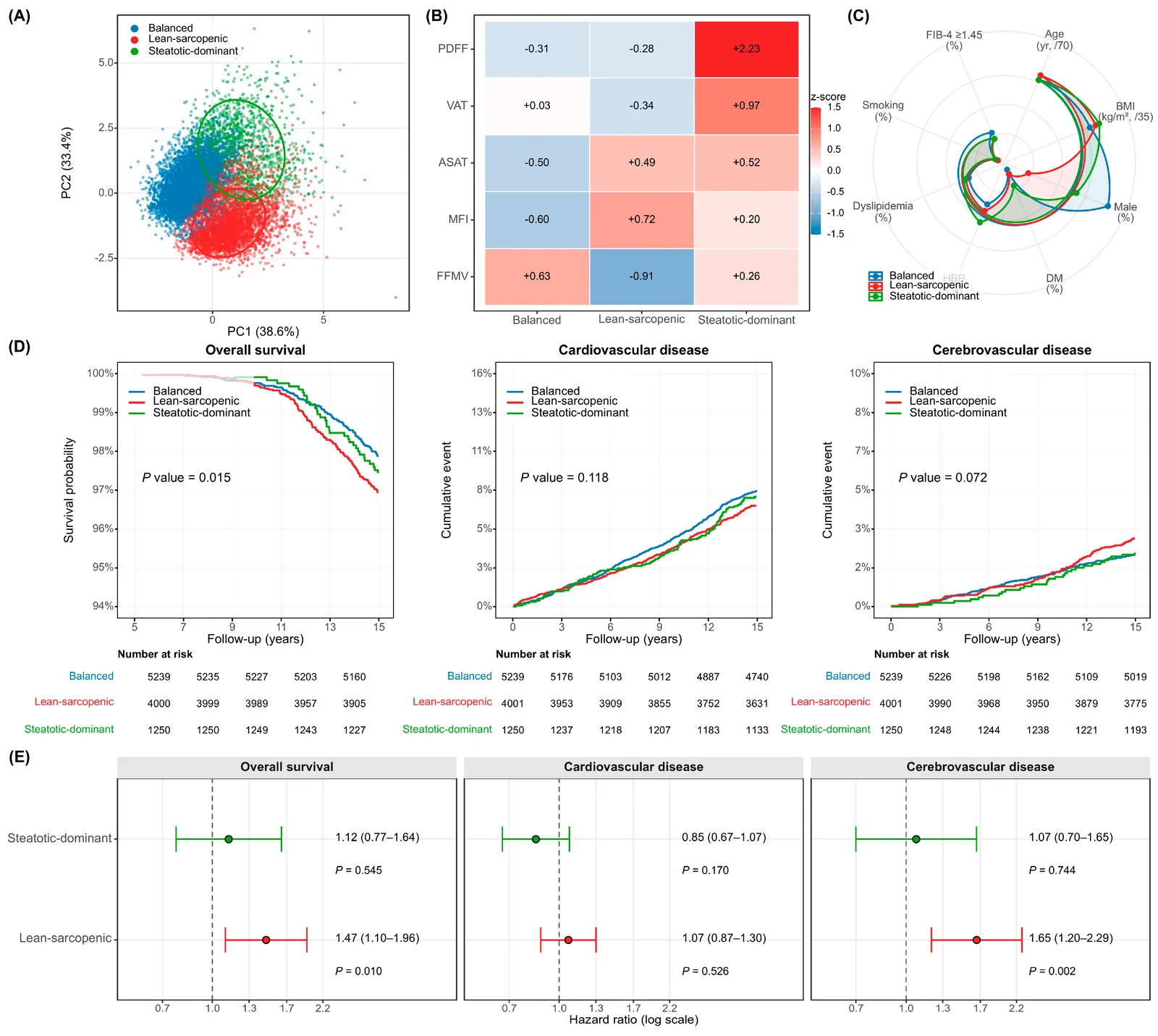
MRI-defined body-composition phenotypes and prognosis in MASLD. (**A**) Principal component plot of the three k-means clusters. (**B**) Cluster profiles across MRI body-composition parameters. (**C**) Baseline clinical characteristics by cluster. (**D**) Kaplan–Meier and cumulative-incidence curves for clinical outcomes. (**E**) Fully adjusted hazard ratios for the lean-sarcopenic and steatotic-dominant clusters, with the balanced cluster as the reference. DM, diabetes mellitus; BMI, body mass index; FIB-4, fibrosis-4 index; PDFF, proton density fat fraction; VAT, visceral adipose tissue; ASAT, abdominal subcutaneous adipose tissue; MFI, muscle fat infiltration; FFMV, fat-free muscle volume.

Clustering based on PDFF, MFI, and FFMV separated individuals in a reduced two-dimensional space defined by the first two principal components (Figure 5A). Clusters were defined by distinct combinations of liver fat and muscle composition. The balanced group showed lower PDFF and MFI with higher FFMV, the lean-sarcopenic group showed higher MFI and lower FFMV at comparable PDFF levels, and the steatotic-dominant group showed higher PDFF with relatively preserved muscle volume. Consistent with these profiles, PDFF was highest in the steatotic-dominant group, whereas MFI was highest and FFMV lowest in the lean-sarcopenic group (Figure 5B, Supplementary Table S6).

Clinical characteristics also differed across clusters. The steatotic-dominant group showed higher BMI and diabetes prevalence, whereas the lean-sarcopenic group was older and had a higher prevalence of hypertension. Sex distribution also varied, with the balanced group predominantly male and the lean-sarcopenic group predominantly female (Figure 5C, Supplementary Table S6).

In outcome analyses, the lean-sarcopenic group showed higher risk of all-cause mortality (adjusted HR 1.47, 95% CI 1.10–1.96; *p* = 0.010) and cerebrovascular disease (adjusted HR 1.65, 95% CI 1.20–2.29; *p* = 0.002) compared with the Balanced reference. Cardiovascular risk did not differ significantly after adjustment. (Figure 5D,E, Supplementary Table S7).

Taken together, clustering identified a lean-sarcopenic phenotype with a distinctly higher risk of mortality and cerebrovascular disease, highlighting the prognostic relevance of muscle composition within MASLD.

### 3.7. Sensitivity Analysis

In a sensitivity analysis using sex-specific standardization of PDFF, MFI, and FFMV, the three body-composition phenotypes remained distinguishable, with comparable sex distributions across clusters (men, 64.1–64.9%). However, the associations of the lean-sarcopenic phenotype with all-cause mortality (adjusted HR 1.23, 95% CI 0.95–1.60; *p* = 0.113) and cerebrovascular disease (adjusted HR 1.14, 95% CI 0.85–1.52; *p* = 0.384) were attenuated and no longer statistically significant. These findings suggest that sex-related differences in muscle composition may have contributed to the outcome associations observed in the original clustering analysis (Supplementary Table S8).

In a sensitivity analysis restricted to participants with FIB-4 < 1.45 (n = 7849), the principal muscle-related associations were largely maintained. Higher MFI remained associated with increased all-cause mortality (adjusted HR 1.10, 95% CI 1.03–1.19; *p* = 0.007), while greater FFMV remained associated with lower cardiovascular risk (adjusted HR 0.93, 95% CI 0.87–0.99; *p* = 0.020). The association between MFI and cerebrovascular disease was similar in magnitude but no longer statistically significant (adjusted HR 1.08, 95% CI 0.99–1.18; *p* = 0.102) (Supplementary Table S9).

In Fine–Gray competing-risk analyses treating death as a competing event, the principal associations were broadly consistent with those of the primary Cox models. Greater FFMV remained associated with lower cardiovascular risk (subdistribution HR 0.93, 95% CI 0.88–0.98; *p* = 0.004), while VAT was positively associated with cardiovascular disease (subdistribution HR 1.06, 95% CI 1.01–1.11; *p* = 0.021). Higher MFI remained associated with cerebrovascular disease (subdistribution HR 1.09, 95% CI 1.01–1.17; *p* = 0.026) (Supplementary Table S10).

## 4. Discussion

The principal finding of this study is that MRI-derived body-composition compartments showed distinct, outcome-specific associations with clinical outcomes in individuals with MASLD. Muscle-related parameters were associated with all-cause mortality and cerebrovascular disease, whereas visceral adiposity and muscle volume showed complementary associations with cardiovascular outcomes. These findings suggest that integrated assessment of hepatic, adipose, and skeletal-muscle compartments might provide a broader characterization of metabolic health than liver fat assessment alone. Instead, the most consistent signals arose from muscle-related parameters and, for cardiovascular outcomes, from visceral adiposity (Figure 1 and Figure 2). This reframes the prognostic structure of MASLD away from a liver-centered model toward a multi-compartment phenotype in which different tissues contribute differently depending on the outcome of interest.

This pattern is consistent with prior observations that liver fat alone is a weak predictor of mortality in population-based cohorts, whereas measures reflecting tissue distribution and quality provide stronger signals [15,21]. The present analysis extends these findings by directly comparing hepatic, adipose, and muscle compartments within the same individuals and by evaluating multiple clinically significant outcomes (Figure 1 and Figure 2, Supplementary Tables S1–S3). The resulting structure is not hierarchical but distributed. Mortality and cerebrovascular risk were more closely associated with muscle-related measures, whereas cardiovascular disease presented contributions from both visceral adiposity and muscle. In particular, Linge et al. [15]. primarily focused on the association between adverse muscle composition and all-cause mortality in NAFLD. Our study extends these findings by examining cardiovascular and cerebrovascular outcomes alongside all-cause mortality and by evaluating the outcome-specific contributions of hepatic fat, abdominal adiposity, and skeletal-muscle composition within an integrated analytical framework.

At the same time, the adipose compartment showed a more outcome-specific role. Visceral fat showed a consistent association with cardiovascular disease and contributed more strongly to risk discrimination at the population level. This pattern is consistent with prior work demonstrating depot-specific associations between adipose tissue and cardiometabolic disease, where visceral fat shows stronger association with vascular outcomes than subcutaneous fat [22,23]. Therefore, the present results support a model in which adipose and muscle compartments contribute complementary, rather than interchangeable, information.

The findings for PDFF should be interpreted with caution. The significant nonlinear association between liver PDFF and all-cause mortality observed in the restricted cubic spline analysis should be distinguished from the nonsignificant association in the linear Cox model. This difference suggests a non-monotonic relationship that may not be adequately represented by a single linear effect estimate. The absence of a consistent independent association does not suggest that hepatic steatosis is not significant. Rather, within a cohort already enriched for metabolic dysfunction, differences in liver fat did not appear to explain residual variation in long-term outcomes once other compartments were taken into account. This distinction is illustrated by the phenotype analysis, where the group with the highest liver fat did not exhibit the highest risk, while a phenotype characterized by less favorable muscle composition showed higher mortality and cerebrovascular risk (Figure 5, Supplementary Tables S6 and S7). In this context, liver fat appears to function more as a marker of disease entry than of downstream risk stratification. The nonlinear association between liver PDFF and all-cause mortality, together with the inverse association between PDFF and cardiovascular disease, may partly present differences in underlying fibrosis severity, as hepatic fat content can decrease with advancing fibrosis. Therefore, these findings should not be interpreted as indicating a protective effect of hepatic steatosis. Furthermore, the MRI-derived parameters were correlated with one another and with conventional anthropometric measures, including BMI. Although mutual adjustment was performed to estimate compartment-specific associations, potential collinearity and the influence of correlated metabolic characteristics should be considered when interpreting the results. In particular, the inverse association between liver PDFF and cardiovascular disease should not be interpreted as indicating a protective effect of hepatic steatosis.

The discrimination analyses further demonstrated that the relative contributions of MRI-derived body-composition compartments differed across outcomes. Models incorporating muscle parameters showed greater discrimination for all-cause mortality, whereas abdominal adiposity models performed better for cardiovascular and numerically for cerebrovascular outcomes. However, the absolute discrimination of these MRI-only models was modest, and their incremental value beyond established clinical risk factors remains uncertain. Accordingly, these findings should be interpreted as comparisons of imaging-derived information rather than evidence of clinically validated prediction models.

From a clinical perspective, these findings support a shift toward integrated interpretation of abdominal MRI in MASLD, where simultaneous assessment of hepatic, adipose, and muscle compartments might provide a more comprehensive representation of risk than liver fat alone. Rather than replacing PDFF, this approach emphasizes its role within an integrated phenotypic framework. This study is based on a large MRI-characterized cohort and a comprehensive analytical framework and uses joint modelling, progressive adjustment and complementary analyses to support the significance of the findings. An important consideration for clinical translation is that thigh-derived MFI and FFMV require extended body-composition imaging and are not routinely available from standard clinical abdominal or liver MRI examinations. Although these measurements provide quantitative information regarding skeletal-muscle quality and quantity, their direct implementation in routine hepatology practice may therefore be limited. Measurements of paraspinal or psoas muscle composition obtained from conventional abdominal imaging may represent more accessible alternatives. However, further studies are required to determine whether these regional muscle measures provide prognostic information comparable to that of quantitative thigh-derived parameters.

Several limitations should be considered. First, the observational design precludes causal inference. Second, the temporal alignment between the initial UK Biobank assessment and subsequent MRI acquisition warrants careful consideration. Follow-up was calculated from the initial recruitment assessment rather than the imaging visit, which may introduce immortal-time bias and temporal misclassification of clinical events. Consequently, the observed associations should be interpreted cautiously and should not be regarded as definitive evidence of prospective prognostic effects following MRI acquisition. Future studies incorporating imaging-anchored follow-up and contemporaneously assessed clinical covariates are required to validate these findings. Third, the UK Biobank cohort is relatively healthy and predominantly of European ancestry, which limit generalizability. Fourth, outcomes were identified through linked administrative data rather than adjudicated review. Fifth, imaging was performed at a single time point, thereby limiting the ability to assess longitudinal change. Another limitation concerns the operational definition of hepatic steatosis. We used an FLI threshold of ≥60 rather than MRI-PDFF to identify participants with presumed hepatic steatosis. Although FLI is an established non-invasive surrogate marker, it does not directly quantify hepatic fat and may classify some individuals differently from MRI-based criteria. Consequently, the findings should be interpreted within the context of an FLI-defined MASLD population, and their generalizability to individuals with MRI-confirmed hepatic steatosis requires further investigation. The clustering-derived phenotypes should also be interpreted cautiously because absolute muscle volume is influenced by sex and body size. Although sex and BMI were included in the multivariable models, the associations between the lean-sarcopenic phenotype and clinical outcomes were attenuated following sex-specific standardization, suggesting that sex-related differences contributed to the original clustering results. Furthermore, muscle strength was not assessed, precluding a formal diagnosis of sarcopenia. The exclusion of visceral adiposity from clustering and the absence of external validation further limit the interpretation and generalizability of these exploratory phenotypes. Although the sensitivity analysis restricted to participants with FIB-4 < 1.45 yielded broadly similar findings, histological fibrosis staging and imaging-based liver stiffness measurements were unavailable. Therefore, residual confounding by underlying liver fibrosis cannot be excluded. Furthermore, the discrimination analyses were restricted to MRI-derived parameters and yielded modest C-indices. Therefore, the incremental predictive value and clinical utility of these imaging measures beyond established clinical risk factors remain to be determined. Additionally, the use of different measurement scales across MRI-derived parameters limits direct comparisons of their effect magnitudes. Although mutually adjusted models were used, correlations among body-composition measures and BMI may have influenced the estimated associations. Physical activity and socioeconomic status were not included in the multivariable models and may have contributed to residual confounding. Furthermore, reverse causation cannot be excluded because sensitivity analyses excluding early clinical events were not performed. The proportional-hazards assumption could not be formally verified, representing an additional limitation of the Cox regression analyses.

## 5. Conclusions

In summary, MRI-derived muscle composition and visceral adiposity showed outcome-specific associations with all-cause mortality and cardiovascular and cerebrovascular outcomes in individuals classified as having MASLD. Muscle-related parameters were particularly associated with mortality and cerebrovascular outcomes, whereas visceral adiposity contributed to cardiovascular risk. These findings highlight the potential value of integrated body-composition assessment while emphasizing the need for further validation in imaging-anchored longitudinal studies.

## Figures and Tables

**Table 1:** Baseline clinical characteristics.

Variable	Overall N = 24,976	No SLD N = 11,909	MASLD N = 10,490	MetALD N = 2577	*p* Value
**Sex**					<0.001
Female	13,382 (53.6%)	8955 (75.2%)	3723 (35.5%)	704 (27.3%)	
Male	11,594 (46.4%)	2954 (24.8%)	6767 (64.5%)	1873 (72.7%)	
**Age**	54.52 ± 7.43	53.50 ± 7.44	54.91 ± 7.31	57.65 ± 6.85	<0.001
**Smoking frequency**					<0.001
Current	1501 (6.0%)	637 (5.3%)	661 (6.3%)	203 (7.9%)	
Never	15,598 (62.5%)	7945 (66.7%)	6442 (61.4%)	1211 (47.0%)	
Previous	7877 (31.5%)	3327 (27.9%)	3387 (32.3%)	1163 (45.1%)	
**Body mass index (kg/m^2^)**	26.19 ± 4.00	23.48 ± 2.24	28.86 ± 3.71	27.90 ± 3.19	<0.001
**Waist circumference (cm)**	86.80 ± 12.23	77.40 ± 7.09	95.49 ± 9.37	94.90 ± 8.85	<0.001
**Type 2 diabetes**	1388 (5.6%)	215 (1.8%)	990 (9.4%)	183 (7.1%)	<0.001
**Dyslipidemia**	6559 (26.3%)	1937 (16.3%)	3608 (34.4%)	1014 (39.3%)	<0.001
**Hypertension**	8092 (32.4%)	2444 (20.5%)	4459 (42.5%)	1189 (46.1%)	<0.001
**ALT (U/L)**	22.39 ± 13.86	17.27 ± 8.20	27.02 ± 16.11	27.26 ± 16.21	<0.001
**AST (U/L)**	25.42 ± 9.95	23.56 ± 7.00	26.99 ± 11.56	27.66 ± 12.59	<0.001
**GGT (U/L)**	31.84 ± 30.37	20.93 ± 11.54	40.71 ± 37.18	46.13 ± 40.31	<0.001
**Total bilirubin (mg/dL)**	0.55 ± 0.27	0.55 ± 0.27	0.55 ± 0.27	0.57 ± 0.27	<0.001
**Albumin (g/L)**	4.54 ± 0.25	4.54 ± 0.25	4.54 ± 0.25	4.56 ± 0.25	0.002
**Platelet (10^9^/L)**	250.82 ± 56.17	251.86 ± 55.38	251.02 ± 57.50	245.23 ± 54.01	<0.001
**Liver PDFF (%)**	4.68 ± 4.71	2.90 ± 2.44	6.35 ± 5.67	6.09 ± 5.32	<0.001
**Visceral adiposity**					
Visceral adipose tissue (VAT) (L)	3.51 ± 2.17	2.10 ± 1.14	4.81 ± 2.09	4.75 ± 2.00	<0.001
Abdominal fat ratio	0.49 ± 0.11	0.46 ± 0.11	0.52 ± 0.10	0.50 ± 0.10	<0.001
Total abdominal adipose tissue index (L/m^2^)	3.58 ± 1.56	2.83 ± 1.16	4.33 ± 1.59	3.96 ± 1.42	<0.001
**Subcutaneous fat**					
Abdominal subcutaneous adipose tissue (ASAT) (L)	6.70 ± 3.04	5.69 ± 2.38	7.79 ± 3.33	6.93 ± 2.91	<0.001
Total trunk fat volume (L)	10.22 ± 4.37	7.80 ± 3.05	12.61 ± 4.27	11.69 ± 3.89	<0.001
**Myosteatosis**					
Muscle fat infiltration (MFI) (%)	7.20 ± 1.81	6.85 ± 1.57	7.51 ± 1.97	7.58 ± 1.86	<0.001
Anterior thigh MFI (left) (%)	7.36 ± 1.85	7.04 ± 1.62	7.64 ± 2.02	7.70 ± 1.88	<0.001
Anterior thigh MFI (right) (%)	7.05 ± 1.85	6.67 ± 1.59	7.38 ± 2.00	7.46 ± 1.94	<0.001
Posterior thigh MFI (left) (%)	10.90 ± 2.34	10.42 ± 2.06	11.31 ± 2.49	11.46 ± 2.42	<0.001
Posterior thigh MFI (right) (%)	10.70 ± 2.35	10.22 ± 2.07	11.10 ± 2.51	11.27 ± 2.46	<0.001
**Muscle volume**					
Total thigh FFMV (L)	10.10 ± 2.54	8.93 ± 2.05	11.17 ± 2.51	11.18 ± 2.33	<0.001
Anterior thigh FFMV (left) (L)	1.70 ± 0.49	1.49 ± 0.40	1.88 ± 0.49	1.88 ± 0.45	<0.001
Anterior thigh FFMV (right) (L)	1.70 ± 0.49	1.50 ± 0.40	1.89 ± 0.49	1.88 ± 0.45	<0.001
Posterior thigh FFMV (left) (L)	3.33 ± 0.80	2.96 ± 0.64	3.68 ± 0.79	3.69 ± 0.74	<0.001
Posterior thigh FFMV (right) (L)	3.38 ± 0.82	2.99 ± 0.65	3.73 ± 0.80	3.73 ± 0.75	<0.001
**Weight-to-muscle ratio (kg/L)**	7.56 ± 1.32	7.49 ± 1.17	7.68 ± 1.46	7.44 ± 1.28	<0.001

Values are presented as n (%) for categorical variables and mean ± standard deviation for continuous variables. *p* values were calculated using Pearson’s chi-square test for categorical variables and the Kruskal–Wallis test for continuous variables. SLD, steatotic liver disease; MASLD, metabolic dysfunction-associated steatotic liver disease; MetALD, MASLD with increased alcohol intake; AST, aspartate aminotransferase; ALT, alanine aminotransferase; GGT, gamma-glutamyl transferase; PDFF, proton density fat fraction; FFMV, fat-free muscle volume.

**Table 2:** Associations of abdominal and muscle composition parameters with clinical outcomes in the MASLD cohort.

**Overall Survival**												
**Variable**	**Unadjusted**			**Model 1**			**Model 2**			**Model 3**		
	**HR**	**95% CI**	***p* Value**	**HR**	**95% CI**	***p* Value**	**HR**	**95% CI**	***p* Value**	**HR**	**95% CI**	***p* Value**
**Liver PDFF (%)**	0.99	0.97, 1.01	0.369	0.99	0.96, 1.01	0.297	0.98	0.96, 1.01	0.142	0.98	0.96, 1.01	0.187
**VAT (L)**	1.10	1.05, 1.16	<0.001	1.05	0.98, 1.13	0.166	1.03	0.96, 1.11	0.443	1.03	0.96, 1.11	0.424
**ASAT (L)**	0.98	0.95, 1.01	0.180	1.00	0.95, 1.05	0.884	0.98	0.92, 1.04	0.482	0.98	0.92, 1.04	0.474
**MFI (%)**	1.16	1.11, 1.21	<0.001	1.13	1.06, 1.19	<0.001	1.10	1.03, 1.16	0.003	1.09	1.03, 1.16	0.003
**Total thigh FFMV (L)**	0.97	0.93, 1.01	0.173	0.96	0.88, 1.04	0.303	0.96	0.88, 1.04	0.292	0.96	0.88, 1.04	0.315
**Incidence of cardiovascular disease**												
**Variable**	**Unadjusted**			**Model 1**			**Model 2**			**Model 3**		
	**HR**	**95% CI**	***p* value**	**HR**	**95% CI**	***p* value**	**HR**	**95% CI**	***p* value**	**HR**	**95% CI**	***p* value**
**Liver PDFF (%)**	1.00	0.99, 1.01	0.823	0.99	0.97, 1.00	0.062	0.98	0.96, 0.99	0.007	0.98	0.97, 1.00	0.024
**VAT (L)**	1.14	1.11, 1.17	<0.001	1.13	1.08, 1.18	<0.001	1.06	1.01, 1.11	0.024	1.06	1.01, 1.11	0.020
**ASAT (L)**	0.95	0.93, 0.98	<0.001	0.99	0.96, 1.03	0.588	1.00	0.96, 1.04	0.881	0.99	0.95, 1.03	0.670
**MFI (%)**	1.05	1.02, 1.09	0.003	1.02	0.98, 1.07	0.281	1.00	0.96, 1.05	0.968	1.00	0.95, 1.04	0.851
**Total thigh FFMV (L)**	1.03	1.00, 1.06	0.054	0.91	0.87, 0.96	<0.001	0.93	0.89, 0.98	0.008	0.93	0.88, 0.98	0.005
**Incidence of cerebrovascular disease**												
**Variable**	**Unadjusted**			**Model 1**			**Model 2**			**Model 3**		
	**HR**	**95% CI**	***p* value**	**HR**	**95% CI**	***p* value**	**HR**	**95% CI**	***p* value**	**HR**	**95% CI**	***p* value**
**Liver PDFF (%)**	0.99	0.97, 1.01	0.511	1.00	0.97, 1.02	0.693	0.99	0.96, 1.01	0.382	0.99	0.96, 1.02	0.432
**VAT (L)**	1.09	1.03, 1.15	0.002	1.07	0.99, 1.16	0.110	1.02	0.94, 1.11	0.620	1.03	0.94, 1.12	0.554
**ASAT (L)**	0.94	0.90, 0.98	0.002	0.94	0.89, 1.01	0.076	0.96	0.89, 1.03	0.257	0.95	0.88, 1.03	0.192
**MFI (%)**	1.11	1.06, 1.17	<0.001	1.10	1.03, 1.18	0.007	1.09	1.01, 1.17	0.025	1.08	1.01, 1.17	0.028
**Total thigh FFMV (L)**	0.98	0.93, 1.03	0.402	0.94	0.86, 1.03	0.173	0.96	0.88, 1.05	0.418	0.95	0.87, 1.05	0.311

Model 1: adjusted for age and sex. Model 2: additionally adjusted for metabolic risk factors (BMI, diabetes, dyslipidemia, hypertension, and smoking). Model 3: additionally adjusted for liver biochemical parameters (ALT, AST, GGT, total bilirubin, albumin, and platelet count). HR, hazard ratio; CI, confidence interval; PDFF, proton density fat fraction; VAT, visceral adipose tissue; ASAT, abdominal subcutaneous adipose tissue; MFI, muscle fat infiltration; FFMV, fat-free muscle volume.

## Data Availability

The data analyzed in this study are available from the UK Biobank upon application and approval. The data are not publicly available due to UK Biobank access restrictions. This study was conducted under UK Biobank Application ID 117214.

## Supplementary Materials

The following supporting information can be downloaded at: https://www.mdpi.com/article/10.3390/diagnostics16213487/s1, Figure S1: Study flow diagram; Figure S2: Distribution of MRI body-composition parameters by SLD class; Figure S3: Correlation between MRI body-composition parameters across SLD classification; Figure S4: Partial-correlation network of MRI, anthropometric, and laboratory variables; Figure S5: Clinical outcomes according to SLD classification; Figure S6: Selection of the optimal number of clusters for MASLD phenotyping; Table S1: Association of abdominal and muscle composition parameters with clinical outcomes in the total cohort; Table S2: Association of abdominal and muscle composition parameters with clinical outcomes in participants without SLD; Table S3: Associations of abdominal and muscle composition parameters with clinical outcomes in the MetALD cohort; Table S4: Per-model prognostic performance of MRI models by SLD classification; Table S5: Bootstrap optimism-corrected discrimination (Harrell’s C-index) and calibration slope for nested MRI prognostic models; Table S6: Cluster-level body composition profile and baseline characteristics in the MASLD cohort; Table S7: Associations between body-composition clusters and clinical outcomes in the MASLD cohort; Table S8: Sensitivity analysis of clustering-derived phenotypes after sex-specific standardization in the MASLD cohort; Table S9: Associations of abdominal and muscle composition parameters with clinical outcomes in the MASLD cohort with FIB-4 < 1.45; Table S10: Fine–Gray competing-risk analysis of MRI body-composition parameters and incident cardiovascular and cerebrovascular disease in the MASLD cohort.

## Abbreviations

The following abbreviations are used in this manuscript:

ASAT	Abdominal subcutaneous adipose tissue
BMI	Body mass index
CI	Confidence interval
FFMV	Fat-free muscle volume
HR	Hazard ratio
MASLD	Metabolic dysfunction-associated steatotic liver disease
MetALD	MASLD with increased alcohol intake
MFI	Muscle fat infiltration
MRI	Magnetic resonance imaging
PDFF	Proton density fat fraction
PM	Proportion mediated
SLD	Steatotic liver disease
VAT	Visceral adipose tissue
