# Supplementary material for: MRI-Derived Muscle Composition and Visceral Adiposity in Relation to Mortality and Vascular Outcomes in MASLD
Source: Diagnostics (Basel). 2026 Oct 24;16(21):3487. doi: 10.3390/diagnostics16213487 (PMC13762111; doi:10.3390/diagnostics16213487)
Supplement: Supplementary file 1 [file diagnostics-16-03487-s001.zip › diagnostics-4629783-supplementary.pdf]

Supplementary Information

Supplementary Figures

- **Supplementary Fig. S1.** Study flow diagram.
- **Supplementary Fig. S2.** Distribution of MRI body-composition parameters by SLD class.
- **Supplementary Fig. S3.** Correlation between MRI body-composition parameters across SLD classification.
- **Supplementary Fig. S4.** Partial-correlation network of MRI, anthropometric, and laboratory variables
- **Supplementary Fig. S5.** Clinical outcomes according to SLD classification.
- **Supplementary Fig. S6.** Selection of the optimal number of clusters for MASLD phenotyping.

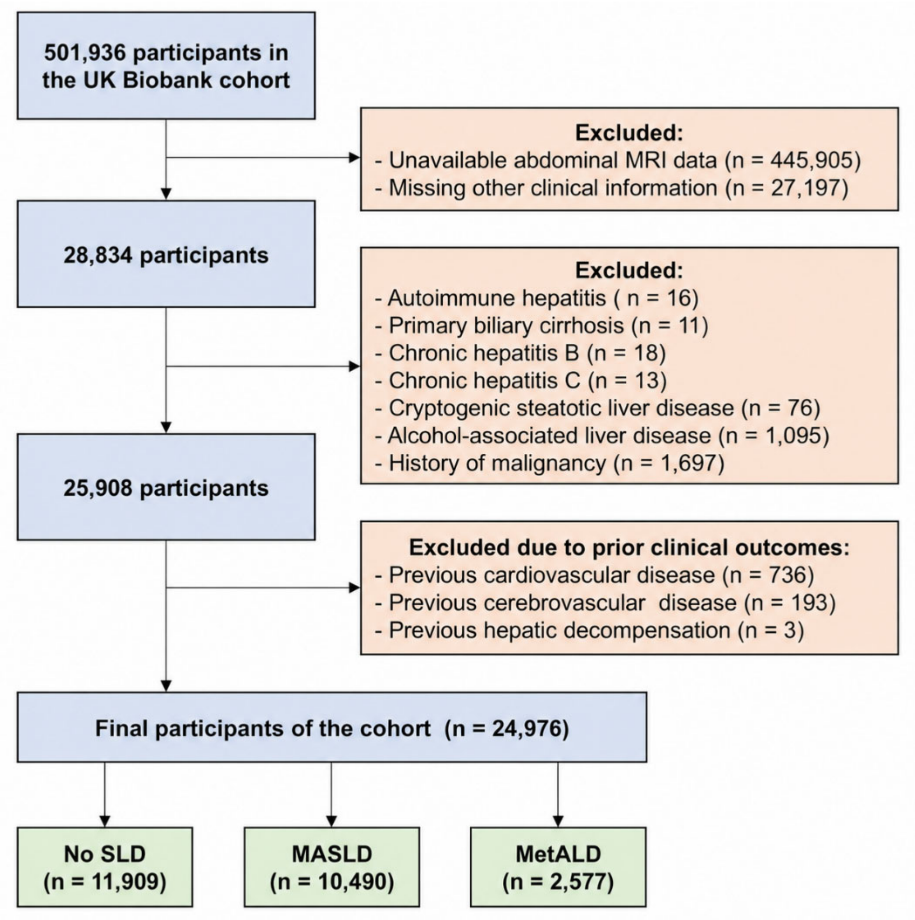

Supplementary Figure S1. Study flow diagram. Flow diagram of participant selection from the UK Biobank cohort to the final analytic sample. Participants were classified as No SLD, MASLD, or MetALD after prespecified exclusions. SLD, steatotic liver disease; MASLD, metabolic dysfunction-associated steatotic liver disease; MetALD, MASLD with increased alcohol intake.

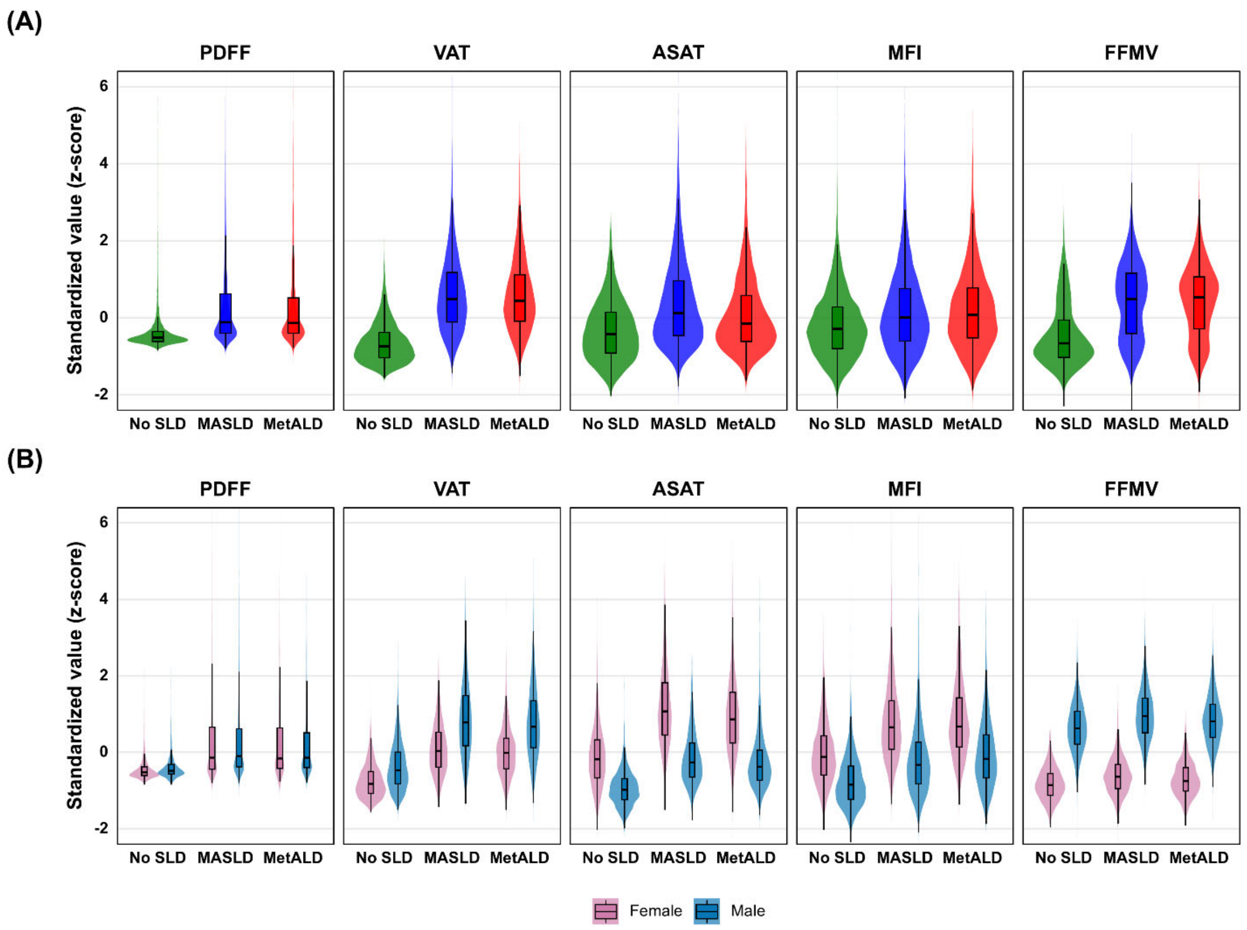

Supplementary Figure S2. Distribution of MRI body-composition parameters by SLD class. (A) Violin plots of standardized PDFF, VAT, ASAT, MFI, and FFMV across the No SLD, MASLD, and MetALD groups. (B) The same distributions further stratified by sex. SLD, steatotic liver disease; MASLD, metabolic dysfunction-associated steatotic liver disease; MetALD, MASLD with increased alcohol intake; PDFF, proton density fat fraction; VAT, visceral adipose tissue; ASAT, abdominal subcutaneous adipose tissue; MFI, muscle fat infiltration; FFMV, fat-free muscle volume.

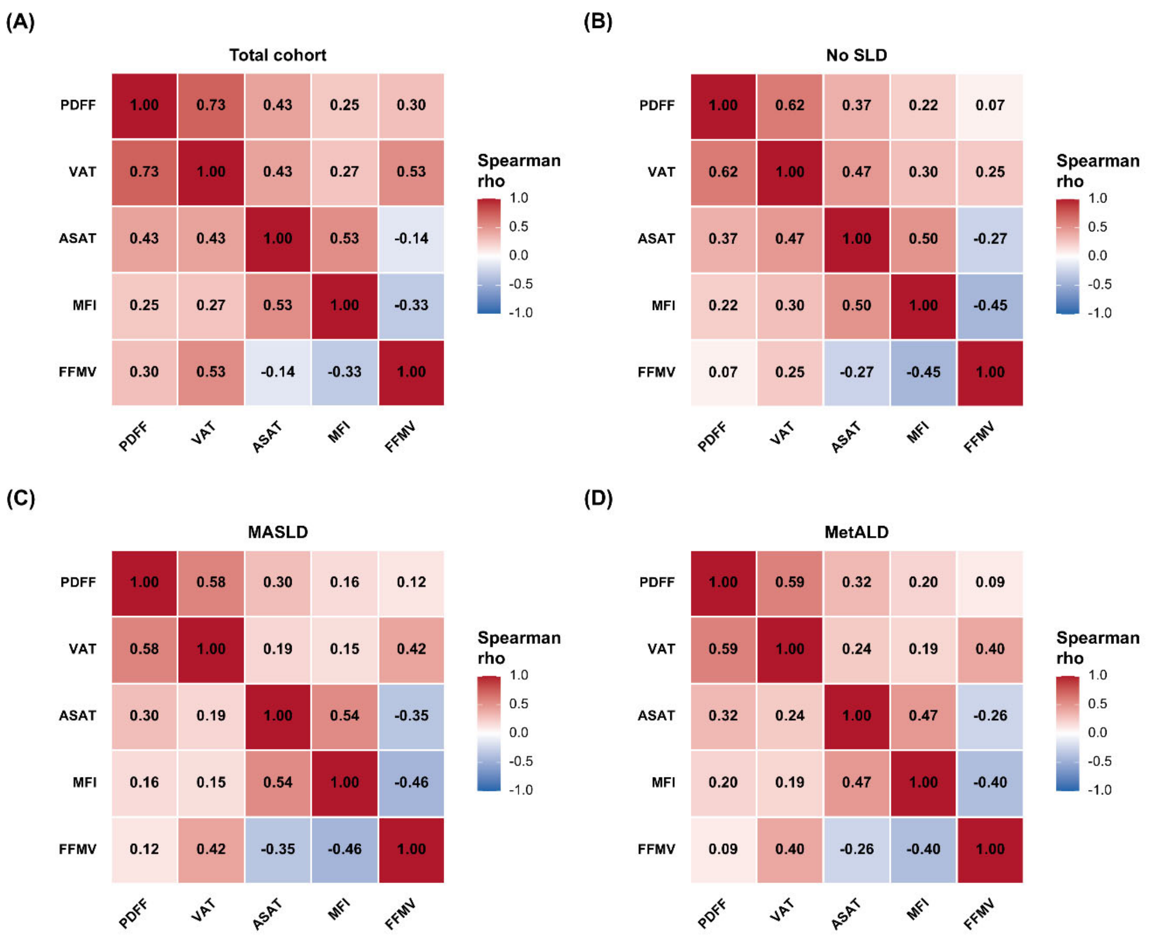

Supplementary Figure S3. Correlation between MRI body-composition parameters across SLD classification. Pairwise Spearman correlation matrices for PDFF, VAT, ASAT, MFI, and FFMV in the total cohort (A), No SLD (B), MASLD (C), and MetALD (D). SLD, steatotic liver disease; MASLD, metabolic dysfunction-associated steatotic liver disease; MetALD, MASLD with increased alcohol intake; PDFF, proton density fat fraction; VAT, visceral adipose tissue; ASAT, abdominal subcutaneous adipose tissue; MFI, muscle fat infiltration; FFMV, fat-free muscle volume.

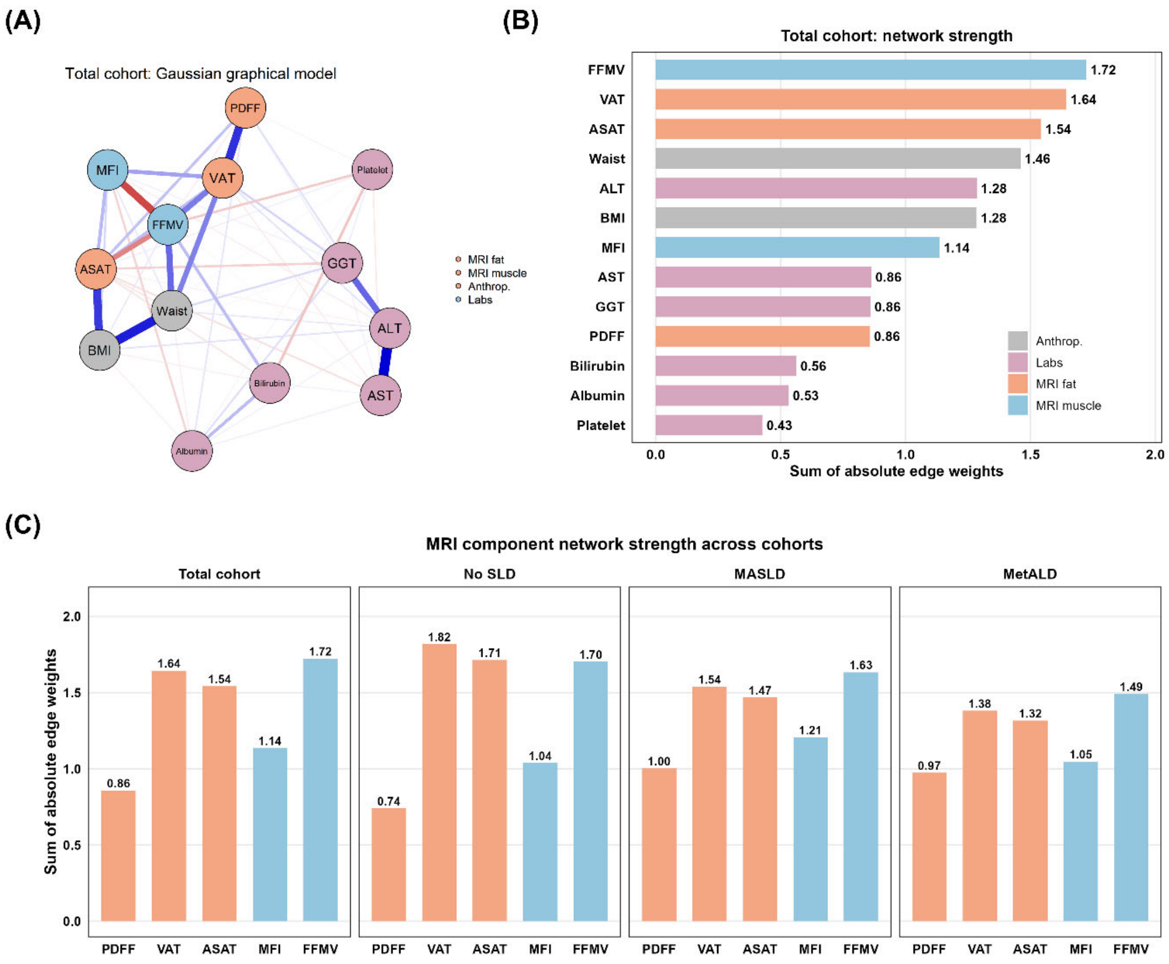

Supplementary Figure S4. Partial-correlation network of MRI, anthropometric, and laboratory variables. (A) Gaussian graphical model in the total cohort. (B) Ranked node strength in the total cohort. (C) MRI component network strength across the total cohort, No SLD, MASLD, and MetALD classification. ALT, alanine aminotransferase; AST, aspartate aminotransferase; BMI, body mass index; GGT, gamma-glutamyl transferase; SLD, steatotic liver disease; MASLD, metabolic dysfunction-associated steatotic liver disease; MetALD, MASLD with increased alcohol intake; PDFF, proton density fat fraction; VAT, visceral adipose tissue; ASAT, abdominal subcutaneous adipose tissue; MFI, muscle fat infiltration; FFMV, fat-free muscle volume.

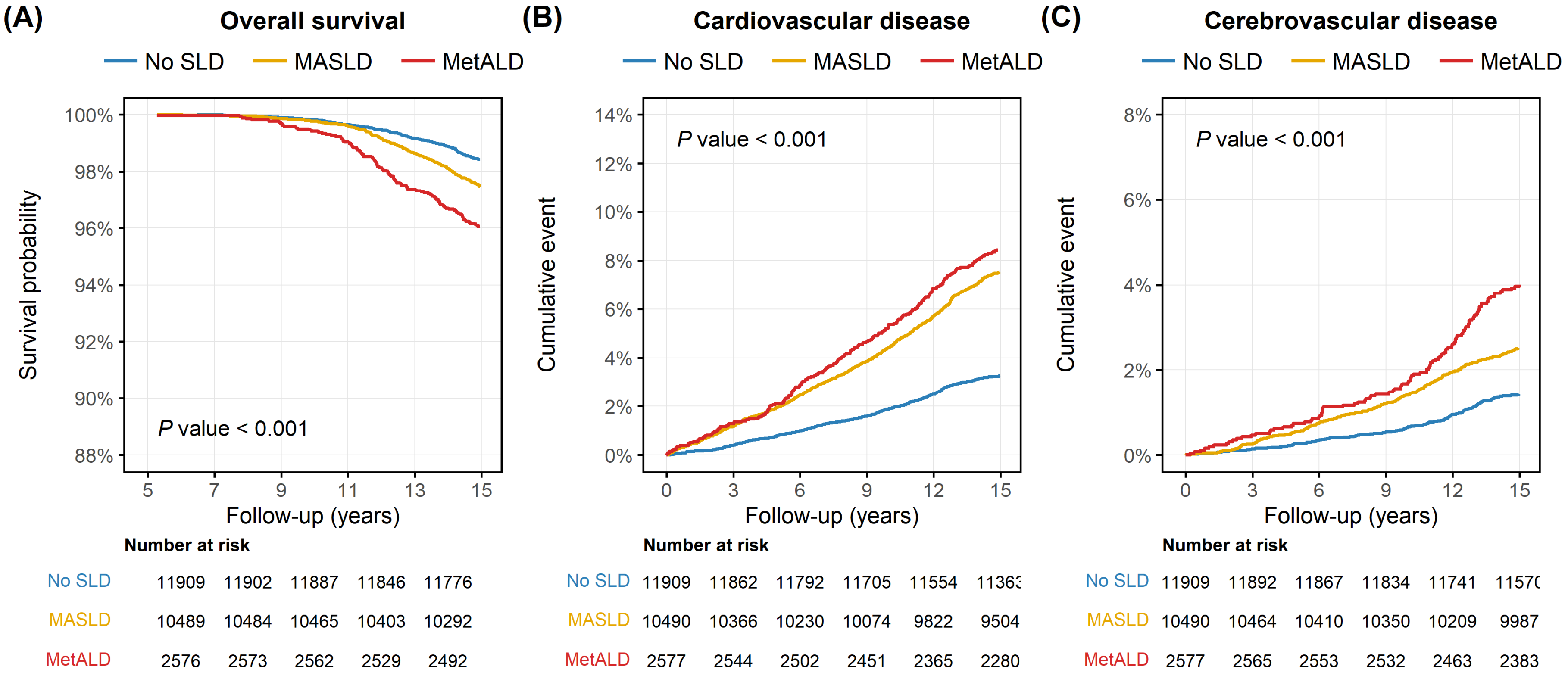

Supplementary Figure S5. Clinical outcomes according to SLD classification. (A) Kaplan-Meier curves for overall survival. (B) Cumulative-incidence curves for incident cardiovascular disease. (C) Cumulative-incidence curves for incident cerebrovascular disease. Numbers at risk are shown below each panel. SLD, steatotic liver disease; MASLD, metabolic dysfunction-associated steatotic liver disease; MetALD, MASLD with increased alcohol intake.

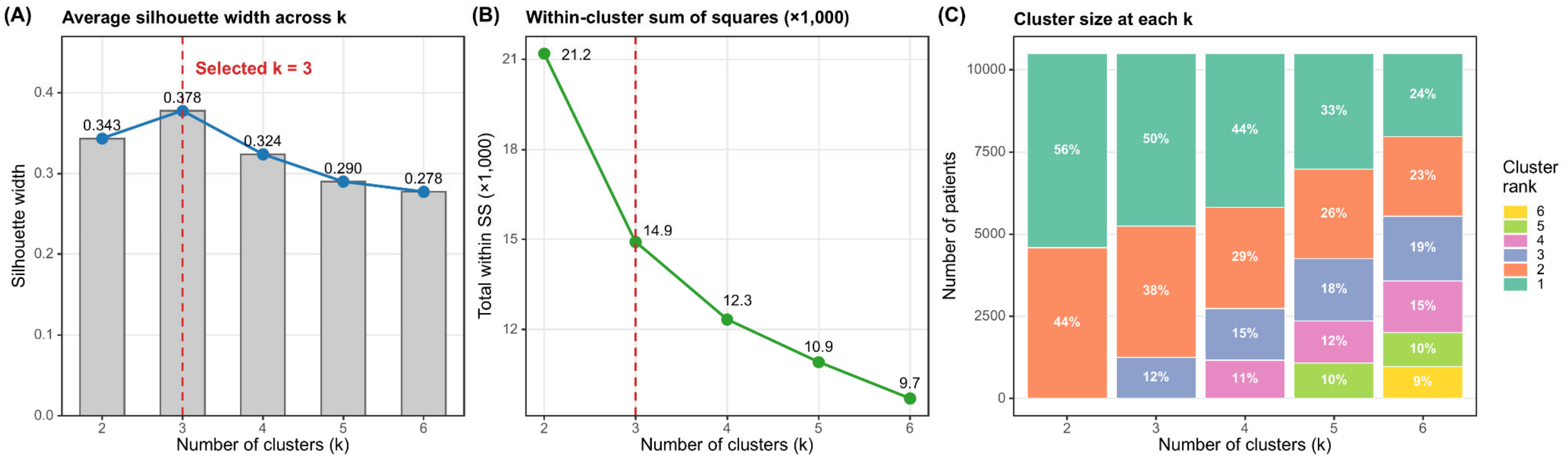

Supplementary Figure S6. Selection of the optimal number of clusters for MASLD phenotyping. (A) Average silhouette width across candidate values of k. (B) Within-cluster sum of squares across candidate values of k. (C) Cluster size distribution for each candidate solution. A three-cluster solution was selected for the MASLD phenotyping analysis.

Supplementary Tables

- **Supplementary Table S1.** Association of abdominal and muscle composition parameters with clinical outcomes in the total cohort.
- **Supplementary Table S2.** Association of abdominal and muscle composition parameters with clinical outcomes in participants without SLD.
- **Supplementary Table S3.** Associations of abdominal and muscle composition parameters with clinical outcomes in the MetALD cohort.
- **Supplementary Table S4.** Per-model prognostic performance of MRI models by SLD classification.
- **Supplementary Table S5.** Bootstrap optimism-corrected discrimination (Harrell's C-index) and calibration slope for nested MRI prognostic models.
- **Supplementary Table S6.** Cluster-level body composition profile and baseline characteristics in the MASLD cohort.
- **Supplementary Table S7.** Associations between body-composition clusters and clinical outcomes in the MASLD cohort
- **Supplementary Table S8.** Sensitivity analysis of clustering-derived phenotypes after sex-specific standardization in the MASLD cohort
- **Supplementary Table S9.** Associations of abdominal and muscle composition parameters with clinical outcomes in the MASLD cohort with FIB-4 < 1.45
- **Supplementary Table S10.** Fine–Gray competing-risk analysis of MRI body-composition parameters and incident cardiovascular and cerebrovascular disease in the MASLD cohort.

Supplementary Table S1. Association of abdominal and muscle composition parameters with clinical outcomes in the total cohort.

Variable	Unadjusted			Model 1			Model 2			Model 3		
	HR	95% CI	*P* value	HR	95% CI	*P* value	HR	95% CI	*P* value	HR	95% CI	*P* value
**Overall survival**												
**Liver PDFF (%)**	1.02	1.01, 1.03	0.006	1.00	0.98, 1.02	0.870	0.99	0.97, 1.01	0.463	1.00	0.98, 1.02	0.700
**VAT (L)**	1.15	1.12, 1.19	< 0.001	1.05	0.99, 1.11	0.095	1.02	0.96, 1.08	0.549	1.02	0.96, 1.08	0.522
**ASAT (L)**	0.99	0.96, 1.01	0.390	0.98	0.95, 1.02	0.417	0.96	0.91, 1.00	0.069	0.95	0.91, 1.00	0.051
**MFI (%)**	1.18	1.14, 1.22	< 0.001	1.13	1.08, 1.18	< 0.001	1.10	1.05, 1.15	< 0.001	1.10	1.05, 1.15	< 0.001
**Total thigh FFMV (L)**	1.05	1.02, 1.08	0.002	0.99	0.93, 1.05	0.738	0.98	0.92, 1.04	0.512	0.98	0.93, 1.05	0.596
**Incidence of cardiovascular disease**												
**Variable**	**Unadjusted**			**Model 1**			**Model 2**			**Model 3**		
	**HR**	**95% CI**	***P* value**	**HR**	**95% CI**	***P* value**	**HR**	**95% CI**	***P* value**	**HR**	**95% CI**	***P* value**
**Liver PDFF (%)**	1.03	1.02, 1.04	< 0.001	0.99	0.98, 1.00	0.058	0.98	0.97, 1.0	0.006	0.98	0.97, 1.00	0.013
**VAT (L)**	1.21	1.19, 1.23	< 0.001	1.13	1.09, 1.17	< 0.001	1.04	1.00, 1.09	0.030	1.04	1.00, 1.08	0.033
**ASAT (L)**	1.00	0.98, 1.01	0.729	1.01	0.99, 1.04	0.285	1.01	0.98, 1.04	0.517	1.01	0.98, 1.04	0.591
**MFI (%)**	1.11	1.08, 1.14	< 0.001	1.04	1.00, 1.07	0.039	1.02	0.98, 1.05	0.396	1.01	0.98, 1.05	0.522
**Total thigh FFMV (L)**	1.10	1.08, 1.12	< 0.001	0.92	0.89, 0.96	< 0.001	0.94	0.91, 0.98	0.005	0.95	0.91, 0.99	0.007
**Incidence of cerebrovascular disease**												
**Variable**	**Unadjusted**			**Model 1**			**Model 2**			**Model 3**		
	**HR**	**95% CI**	***P* value**	**HR**	**95% CI**	***P* value**	**HR**	**95% CI**	***P* value**	**HR**	**95% CI**	***P* value**
**Liver PDFF (%)**	1.01	0.99, 1.03	0.216	0.99	0.97, 1.01	0.253	0.98	0.96, 1.00	0.091	0.98	0.96, 1.01	0.154
**VAT (L)**	1.14	1.10, 1.18	< 0.001	1.08	1.01, 1.15	0.016	1.03	0.96, 1.09	0.455	1.03	0.96, 1.10	0.432
**ASAT (L)**	0.97	0.94, 1.00	0.033	0.97	0.92, 1.01	0.134	0.97	0.92, 1.03	0.329	0.97	0.92, 1.02	0.267
**MFI (%)**	1.14	1.09, 1.18	< 0.001	1.09	1.03, 1.15	0.002	1.07	1.01, 1.13	0.015	1.07	1.01, 1.13	0.019
**Total thigh FFMV (L)**	1.05	1.02, 1.08	0.003	0.98	0.92, 1.05	0.539	1.00	0.94, 1.07	0.904	1.01	0.94, 1.08	0.883

Model 1: adjusted for age and sex.
Model 2: additionally adjusted for metabolic risk factors (BMI, diabetes, dyslipidemia, hypertension, and smoking).
Model 3: additionally adjusted for liver biochemical parameters (ALT, AST, GGT, total bilirubin, albumin, and platelet count).
HR, hazard ratio; CI, confidence interval; PDFF, proton density fat fraction; VAT, visceral adipose tissue; ASAT, abdominal subcutaneous adipose tissue; MFI, muscle fat infiltration; FFMV, fat-free muscle volume.

Supplementary Table S2. Association of abdominal and muscle composition parameters with clinical outcomes in participants without SLD.

Overall survival												
Variable	Unadjusted			Model 1			Model 2			Model 3		
	HR	95% CI	*P* value	HR	95% CI	*P* value	HR	95% CI	*P* value	HR	95% CI	*P* value
**Liver PDFF (%)**	1.02	0.97, 1.07	0.507	1.02	0.97, 1.09	0.407	1.02	0.96, 1.08	0.521	1.02	0.96, 1.08	0.518
**VAT (L)**	1.07	0.95, 1.19	0.259	0.95	0.80, 1.14	0.593	0.93	0.78, 1.12	0.459	0.93	0.78, 1.11	0.432
**ASAT (L)**	0.92	0.87, 0.98	0.006	0.96	0.87, 1.05	0.396	0.96	0.86, 1.07	0.481	0.96	0.86, 1.07	0.448
**MFI (%)**	1.15	1.08, 1.23	< 0.001	1.09	1.00, 1.20	0.060	1.08	0.99, 1.19	0.100	1.09	0.99, 1.20	0.093
**Total thigh FFMV (L)**	1.00	0.94, 1.07	0.982	1.04	0.92, 1.16	0.557	1.04	0.92, 1.17	0.528	1.05	0.93, 1.19	0.408
**Incidence of cardiovascular disease**												
Variable	Unadjusted			Model 1			Model 2			Model 3		
	HR	95% CI	*P* value	HR	95% CI	*P* value	HR	95% CI	*P* value	HR	95% CI	*P* value
**Liver PDFF (%)**	1.04	1.01, 1.08	0.003	1.01	0.97, 1.05	0.578	1.01	0.97, 1.04	0.762	1.00	0.97, 1.04	0.802
**VAT (L)**	1.31	1.21, 1.40	< 0.001	1.17	1.04, 1.31	0.01	1.05	0.93, 1.18	0.409	1.04	0.92, 1.17	0.520
**ASAT (L)**	0.95	0.91, 0.99	0.024	1.00	0.93, 1.07	0.985	0.99	0.92, 1.07	0.853	1.00	0.92, 1.08	0.951
**MFI (%)**	1.13	1.07, 1.19	< 0.001	1.04	0.97, 1.12	0.229	1.02	0.95, 1.10	0.562	1.02	0.94, 1.10	0.634
**Total thigh FFMV (L)**	1.04	0.99, 1.09	0.116	0.88	0.80, 0.95	0.003	0.9	0.83, 0.99	0.026	0.92	0.84, 1.00	0.059
**Incidence of cerebrovascular disease**												
Variable	Unadjusted			Model 1			Model 2			Model 3		
	HR	95% CI	*P* value	HR	95% CI	*P* value	HR	95% CI	*P* value	HR	95% CI	*P* value
**Liver PDFF (%)**	1.02	0.97, 1.08	0.479	1.01	0.95, 1.08	0.767	1.00	0.94, 1.07	0.899	1.01	0.95, 1.08	0.796
**VAT (L)**	1.11	0.98, 1.26	0.088	1.09	0.90, 1.33	0.377	1.00	0.82, 1.22	0.972	1.01	0.83, 1.23	0.922
**ASAT (L)**	0.94	0.88, 1.00	0.061	0.94	0.84, 1.04	0.244	0.98	0.87, 1.11	0.792	0.98	0.87, 1.11	0.773
**MFI (%)**	1.13	1.04, 1.22	0.003	1.05	0.94, 1.17	0.422	1.03	0.92, 1.16	0.562	1.02	0.91, 1.15	0.714
**Total thigh FFMV (L)**	0.99	0.91, 1.06	0.697	0.99	0.87, 1.13	0.873	1.05	0.92, 1.21	0.450	1.07	0.93, 1.23	0.372

Model 1: adjusted for age and sex.
Model 2: additionally adjusted for metabolic risk factors (BMI, diabetes, dyslipidemia, hypertension, and smoking).
Model 3: additionally adjusted for liver biochemical parameters (ALT, AST, GGT, total bilirubin, albumin, and platelet count).
HR, hazard ratio; CI, confidence interval; PDFF, proton density fat fraction; VAT, visceral adipose tissue; ASAT, abdominal subcutaneous adipose tissue; MFI, muscle fat infiltration; FFMV, fat-free muscle volume.

Supplementary Table S3. Associations of abdominal and muscle composition parameters with clinical outcomes in the MetALD cohort.

Variable	Unadjusted			Model 1			Model 2			Model 3		
**Overall survival**												
	HR	95% CI	*P* value	HR	95% CI	*P* value	HR	95% CI	*P* value	HR	95% CI	*P* value
**Liver PDFF (%)**	1.02	0.99, 1.05	0.321	1.01	0.98, 1.05	0.533	1.01	0.97, 1.04	0.73	1.02	0.98, 1.06	0.366
**VAT (L)**	1.14	1.05, 1.24	0.001	1.03	0.91, 1.17	0.611	1.00	0.88, 1.14	0.971	1.01	0.89, 1.14	0.913
**ASAT (L)**	0.94	0.88, 1.00	0.061	0.97	0.88, 1.08	0.618	0.93	0.83, 1.05	0.227	0.92	0.82, 1.03	0.160
**MFI (%)**	1.15	1.06, 1.24	0.001	1.16	1.05, 1.28	0.004	1.12	1.00, 1.25	0.045	1.11	0.99, 1.24	0.065
**Total thigh FFMV (L)**	1.05	0.97, 1.13	0.219	0.99	0.87, 1.12	0.831	0.97	0.85, 1.10	0.603	0.98	0.86, 1.11	0.715
**Incidence of cardiovascular disease**												
**Variable**	**Unadjusted**			**Model 1**			**Model 2**			**Model 3**		
	HR	95% CI	*P* value	HR	95% CI	*P* value	HR	95% CI	*P* value	HR	95% CI	*P* value
**Liver PDFF (%)**	0.98	0.96, 1.01	0.230	0.97	0.94, 1.01	0.107	0.97	0.94, 1.00	0.067	0.97	0.94, 1.00	0.084
**VAT (L)**	1.09	1.02, 1.16	0.007	1.06	0.96, 1.16	0.239	1.00	0.90, 1.10	0.977	1.00	0.90, 1.10	0.974
**ASAT (L)**	0.95	0.90, 0.99	0.027	1.01	0.93, 1.08	0.876	1.06	0.97, 1.16	0.172	1.06	0.97, 1.16	0.171
**MFI (%)**	1.06	1.00, 1.14	0.071	1.07	0.98, 1.16	0.144	1.07	0.98, 1.17	0.125	1.07	0.98, 1.17	0.132
**Total thigh FFMV (L)**	1.04	0.98, 1.10	0.162	0.96	0.87, 1.06	0.431	1.02	0.92, 1.13	0.675	1.03	0.93, 1.14	0.570
**Incidence of cerebrovascular disease**												
**Variable**	**Unadjusted**			**Model 1**			**Model 2**			**Model 3**		
	HR	95% CI	*P* value	HR	95% CI	*P* value	HR	95% CI	*P* value	HR	95% CI	*P* value
**Liver PDFF (%)**	0.95	0.90, 0.99	0.028	0.95	0.89, 1.00	0.060	0.94	0.89, 1.00	0.033	0.94	0.88, 0.99	0.031
**VAT (L)**	1.02	0.93, 1.12	0.690	1.00	0.87, 1.15	0.983	0.98	0.85, 1.13	0.748	0.98	0.85, 1.14	0.817
**ASAT (L)**	0.92	0.85, 0.99	0.031	1.00	0.90, 1.12	0.964	1.05	0.92, 1.20	0.477	1.05	0.92, 1.20	0.489
**MFI (%)**	1.06	0.96, 1.17	0.254	1.08	0.96, 1.23	0.207	1.07	0.94, 1.22	0.296	1.07	0.94, 1.22	0.290
**Total thigh FFMV (L)**	1.04	0.96, 1.13	0.363	1.02	0.89, 1.18	0.731	1.06	0.92, 1.22	0.430	1.05	0.91, 1.22	0.478

Model 1: adjusted for age and sex.
Model 2: additionally adjusted for metabolic risk factors (BMI, diabetes, dyslipidemia, hypertension, and smoking).
Model 3: additionally adjusted for liver biochemical parameters (ALT, AST, GGT, total bilirubin, albumin, and platelet count).
HR, hazard ratio; CI, confidence interval; PDFF, proton density fat fraction; VAT, visceral adipose tissue; ASAT, abdominal subcutaneous adipose tissue; MFI, muscle fat infiltration; FFMV, fat-free muscle volume.

Supplementary Table S4. Per-model prognostic performance of MRI models by SLD classification.

Stratum	Outcome	Model	Number	Events	C-index (95% CI)
**Total cohort**	**Overall survival**	PDFF	24,976	684	0.537 (0.516–0.557)
		PDFF + VAT + ASAT	24,976	684	0.607 (0.586–0.628)
		PDFF + MFI + FFMV	24,976	684	0.622 (0.603–0.642)
	**Cardiovascular disease**	PDFF	24,976	1415	0.575 (0.561–0.589)
		PDFF + VAT + ASAT	24,976	1415	0.655 (0.642–0.669)
		PDFF + MFI + FFMV	24,976	1415	0.612 (0.597–0.627)
	**Cerebrovascular disease**	PDFF	24,976	542	0.527 (0.484–0.553)
		PDFF + VAT + ASAT	24,976	542	0.615 (0.594–0.638)
		PDFF + MFI + FFMV	24,976	542	0.601 (0.578–0.626)
**No SLD**	**Overall survival**	PDFF	11,909	223	0.503 (0.472–0.548)
		PDFF + VAT + ASAT	11,909	223	0.567 (0.539–0.606)
		PDFF + MFI + FFMV	11,909	223	0.588 (0.554–0.625)
	**Cardiovascular disease**	PDFF	11,909	392	0.550 (0.522–0.583)
		PDFF + VAT + ASAT	11,909	392	0.623 (0.596–0.653)
		PDFF + MFI + FFMV	11,909	392	0.596 (0.570–0.624)
	**Cerebrovascular disease**	PDFF	11,909	171	0.470 (0.442–0.567)
		PDFF + VAT + ASAT	11,909	171	0.563 (0.523–0.617)
		PDFF + MFI + FFMV	11,909	171	0.558 (0.517–0.602)
**MASLD**	**Overall survival**	PDFF	10,490	337	0.534 (0.473–0.562)
		PDFF + VAT + ASAT	10,490	337	0.572 (0.547–0.604)
		PDFF + MFI + FFMV	10,490	337	0.610 (0.581–0.639)
	**Cardiovascular disease**	PDFF	10,490	804	0.493 (0.486–0.526)
		PDFF + VAT + ASAT	10,490	804	0.607 (0.589–0.626)
		PDFF + MFI + FFMV	10,490	804	0.540 (0.524–0.562)
	**Cerebrovascular disease**	PDFF	10,490	265	0.510 (0.489–0.545)
		PDFF + VAT + ASAT	10,490	265	0.588 (0.559–0.625)
		PDFF + MFI + FFMV	10,490	265	0.570 (0.540–0.609)
**MetALD**	**Overall survival**	PDFF	2,577	124	0.520 (0.484–0.573)
		PDFF + VAT + ASAT	2,577	124	0.609 (0.562–0.663)
		PDFF + MFI + FFMV	2,577	124	0.598 (0.557–0.657)
	**Cardiovascular disease**	PDFF	2,577	219	0.523 (0.490–0.561)
		PDFF + VAT + ASAT	2,577	219	0.596 (0.563–0.639)
		PDFF + MFI + FFMV	2,577	219	0.565 (0.536–0.606)
	**Cerebrovascular disease**	PDFF	2,577	106	0.549 (0.497–0.602)
		PDFF + VAT + ASAT	2,577	106	0.585 (0.543–0.641)
		PDFF + MFI + FFMV	2,577	106	0.587 (0.544–0.642)

MRI, magnetic resonance imaging; SLD, steatotic liver disease; MASLD, metabolic dysfunction–associated steatotic liver disease; MetALD, MASLD with increased alcohol intake; CI, confidence interval; PDFF, proton density fat fraction; VAT, visceral adipose tissue; ASAT, abdominal subcutaneous adipose tissue; MFI, muscle fat infiltration; FFMV, fat-free muscle volume.

Supplementary Table S5. Bootstrap optimism-corrected discrimination (Harrell's C-index) and calibration slope for nested MRI prognostic models.

Cohort	Outcome	Model	Number	Events	C-index (apparent)	C-index (bootstrap-corrected, 95% CI)	Optimism	Calibration slope (bootstrap-corrected)
**Total cohort**	Overall survival	PDFF	24,976	684	0.537	0.535 (0.514-0.557)	0.001	1.631
		PDFF + VAT + ASAT	24,976	684	0.607	0.605 (0.584-0.627)	0.002	0.991
		PDFF + MFI + FFMV	24,976	684	0.622	0.620 (0.599-0.641)	0.002	0.993
	Cardiovascular disease	PDFF	24,976	1,415	0.575	0.575 (0.560-0.589)	0.000	1.035
		PDFF + VAT + ASAT	24,976	1,415	0.655	0.654 (0.640-0.668)	0.001	0.997
		PDFF + MFI + FFMV	24,976	1,415	0.612	0.611 (0.597-0.625)	0.001	0.994
	Cerebrovascular disease	PDFF	24,976	542	0.527	0.524 (0.500-0.549)	0.003	3.273
		PDFF + VAT + ASAT	24,976	542	0.615	0.613 (0.589-0.636)	0.002	0.990
		PDFF + MFI + FFMV	24,976	542	0.601	0.598 (0.574-0.622)	0.003	0.985
**No SLD**	Overall survival	PDFF	11,909	223	0.503	0.493 (0.452-0.534)	0.010	1.711
		PDFF + VAT + ASAT	11,909	223	0.567	0.559 (0.521-0.596)	0.009	0.911
		PDFF + MFI + FFMV	11,909	223	0.588	0.579 (0.542-0.616)	0.009	0.943
	Cardiovascular disease	PDFF	11,909	392	0.550	0.549 (0.520-0.579)	0.001	1.110
		PDFF + VAT + ASAT	11,909	392	0.623	0.619 (0.590-0.648)	0.004	0.983
		PDFF + MFI + FFMV	11,909	392	0.596	0.592 (0.565-0.620)	0.003	0.975
	Cerebrovascular disease	PDFF	11,909	171	0.470	0.459 (0.417-0.501)	0.010	1.117
		PDFF + VAT + ASAT	11,909	171	0.563	0.551 (0.506-0.596)	0.012	0.839
		PDFF + MFI + FFMV	11,909	171	0.558	0.545 (0.503-0.586)	0.014	0.835
**MASLD**	Overall survival	PDFF	10,490	337	0.534	0.527 (0.496-0.558)	0.007	0.316
		PDFF + VAT + ASAT	10,490	337	0.572	0.566 (0.535-0.598)	0.006	0.954
		PDFF + MFI + FFMV	10,490	337	0.610	0.604 (0.574-0.635)	0.005	0.983
	Cardiovascular disease	PDFF	10,490	804	0.493	0.487 (0.467-0.507)	0.006	0.266
		PDFF + VAT + ASAT	10,490	804	0.607	0.605 (0.586-0.625)	0.002	0.991
		PDFF + MFI + FFMV	10,490	804	0.540	0.536 (0.516-0.556)	0.004	0.965
	Cerebrovascular disease	PDFF	10,490	265	0.510	0.501 (0.466-0.536)	0.009	-0.919
		PDFF + VAT + ASAT	10,490	265	0.588	0.580 (0.546-0.614)	0.008	0.938
		PDFF + MFI + FFMV	10,490	265	0.570	0.562 (0.527-0.597)	0.008	0.968
**MetALD**	Overall survival	PDFF	2,577	124	0.520	0.507 (0.455-0.559)	0.013	-10.539
		PDFF + VAT + ASAT	2,577	124	0.609	0.597 (0.549-0.646)	0.012	0.917
		PDFF + MFI + FFMV	2,577	124	0.598	0.587 (0.535-0.638)	0.011	0.954
	Cardiovascular disease	PDFF	2,577	219	0.523	0.517 (0.479-0.554)	0.006	4.000
		PDFF + VAT + ASAT	2,577	219	0.596	0.588 (0.553-0.624)	0.008	0.940
		PDFF + MFI + FFMV	2,577	219	0.565	0.557 (0.520-0.594)	0.008	0.938
	Cerebrovascular disease	PDFF	2,577	106	0.549	0.551 (0.497-0.604)	-0.001	1.202
		PDFF + VAT + ASAT	2,577	106	0.585	0.571 (0.520-0.621)	0.015	0.895
		PDFF + MFI + FFMV	2,577	106	0.587	0.574 (0.521-0.626)	0.013	0.900

Apparent C-index is Harrell's concordance index from the fitted Cox proportional-hazards model within each cohort stratum. The bootstrap-corrected C-index removes the optimism arising from using the same data for model fitting and evaluation, computed from 500 bootstrap resamples using Harrell's procedure. Optimism is the average difference in discrimination between the bootstrap samples and the original data. The calibration slope (ideal = 1.00) is also bootstrap-corrected. Nested models progressively add MRI-derived parameters. C-index, Harrell's concordance index; SLD, steatotic liver disease; MASLD, metabolic dysfunction–associated steatotic liver disease; MetALD, MASLD with increased alcohol intake; PDFF, proton density fat fraction; VAT, visceral adipose tissue; ASAT, abdominal subcutaneous adipose tissue; MFI, muscle fat infiltration; FFMV, fat-free muscle volume. <u>Extreme calibration slope estimates observed in some PDFF-only models may reflect instability in calibration estimation when the underlying prognostic signal is weak. These estimates should be interpreted cautiously, and further validation is required to establish their reliability.</u>

Supplementary Table S6. Cluster-level body composition profile and baseline characteristics in the MASLD cohort.

Variable	Balanced N = 5,239	Lean-sarcopenic N = 4,001	Steatotic-dominant N = 1,250	*P* value
Age (years)	53.8 ± 7.4	56.7 ± 7.0	53.8 ± 7.0	< 0.001
Male, n (%)	5038 (96.2)	893 (22.3)	836 (66.9)	< 0.001
BMI (kg/m²)	27.8 ± 3.0	27.6 ± 3.9	30.8 ± 4.2	< 0.001
Diabetes, n (%)	323 (6.2)	406 (10.1)	261 (20.9)	< 0.001
Hypertension, n (%)	2006 (38.3)	1769 (44.2)	684 (54.7)	< 0.001
Dyslipidemia, n (%)	1733 (33.1)	1420 (35.5)	455 (36.4)	0.015
Current smoker, n (%)	320 (6.1)	236 (5.9)	105 (8.4)	0.005
ALT (U/L)	24.2 [19.3–32.0]	20.5 [16.1–27.1]	31.2 [22.9–43.4]	< 0.001
AST (U/L)	26.0 [22.6–30.1]	23.6 [20.6–27.4]	27.8 [23.3–34.0]	< 0.001
GGT (U/L)	32.3 [24.0–46.1]	26.8 [19.9–40.5]	37.6 [26.9–53.9]	< 0.001
Albumin (g/L)	4.6 ± 0.2	4.5 ± 0.3	4.6 ± 0.2	< 0.001
Platelet (10^9/L)	239.5 ± 53.2	266.7 ± 59.5	249.2 ± 56.4	< 0.001
FIB-4 ≥ 1.45, n (%)	1473 (28.1)	890 (22.2)	281 (22.5)	< 0.001
Liver PDFF (%)	4.6 ± 2.6	4.7 ± 2.9	19.0 ± 5.9	< 0.001
VAT (L)	4.9 ± 1.9	4.1 ± 1.8	6.8 ± 2.2	< 0.001
ASAT (L)	6.1 ± 2.3	9.4 ± 3.3	9.5 ± 3.7	< 0.001
MFI (%)	6.3 ± 1.1	8.9 ± 1.9	7.9 ± 1.8	< 0.001
Total thigh FFMV (L)	12.8 ± 1.7	8.9 ± 1.5	11.8 ± 2.4	< 0.001

Values are presented as mean ± standard deviation for continuous variables, median [interquartile range] for skewed continuous variables (ALT, AST, GGT), and n (%) for categorical variables. P values were derived from one-way ANOVA for normally distributed continuous variables, the Kruskal–Wallis test for non-normally distributed continuous variables, and the chi-square test for categorical variables. Clusters were derived from unsupervised k-means clustering (k = 3) on standardized values of liver PDFF, MFI, and total thigh FFMV within the MASLD cohort. MASLD, metabolic dysfunction–associated steatotic liver disease; BMI, body mass index; ALT, alanine aminotransferase; AST, aspartate aminotransferase; GGT, gamma-glutamyl transferase; FIB-4, Fibrosis-4 index; PDFF, proton density fat fraction; VAT, visceral adipose tissue; ASAT, abdominal subcutaneous adipose tissue; MFI, muscle fat infiltration; FFMV, fat-free muscle volume.

Supplementary Table S7. Associations between body-composition clusters and clinical outcomes in the MASLD cohort

Outcome	Cluster	Number	Events	Crude HR (95% CI)	Crude *P*	Adjusted HR (95% CI)	Adjusted *P*
**All-cause mortality**	**Balanced**	5,239	144	Reference	-	Reference	-
	**Lean-sarcopenic**	4,001	153	1.40 (1.11–1.76)	0.004	1.47 (1.10–1.96)	0.010
	**Steatotic-dominant**	1,250	40	1.17 (0.82–1.66)	0.386	1.12 (0.77–1.64)	0.545
**Cardiovascular disease**	**Balanced**	5,239	429	Reference	-	Reference	-
	**Lean-sarcopenic**	4,001	281	0.85 (0.74–0.99)	0.040	1.07 (0.87–1.30)	0.526
	**Steatotic-dominant**	1,250	94	0.91 (0.73–1.14)	0.430	0.85 (0.67–1.07)	0.170
**Cerebrovascular disease**	**Balanced**	5,239	117	Reference	-	Reference	-
	**Lean-sarcopenic**	4,001	119	1.34 (1.03–1.72)	0.026	1.65 (1.20–2.29)	0.002
	**Steatotic-dominant**	1,250	29	1.04 (0.69–1.56)	0.860	1.07 (0.70–1.65)	0.744

Values are presented as crude and multivariable-adjusted hazard ratios (HRs) with 95% confidence intervals from Cox proportional-hazards models, using the Balanced phenotype as the reference. Models were adjusted for age, sex, body mass index, diabetes, dyslipidemia, hypertension, smoking status, alanine aminotransferase, aspartate aminotransferase, gamma-glutamyl transferase, total bilirubin, albumin, and platelet count. Follow-up was based on the initial UK Biobank recruitment assessment rather than the subsequent MRI examination; therefore, the estimated associations should be interpreted cautiously regarding the temporal alignment between imaging-derived measurements and clinical outcomes. MASLD, metabolic dysfunction-associated steatotic liver disease; HR, hazard ratio; CI, confidence interval; BMI, body mass index; ALT, alanine aminotransferase; AST, aspartate aminotransferase; GGT, gamma-glutamyl transferase.

Supplementary Table S8. Sensitivity analysis of clustering-derived phenotypes after sex-specific standardization in the MASLD cohort

Outcome	Phenotype	Adjusted HR	95% CI	*P* value
Overall survival	Balanced	Reference		
	Lean sarcopenic	1.23	0.95–1.60	0.113
	Steatotic-dominant	1.01	0.69–1.49	0.949
Cardiovascular disease	Balanced	Reference		
	Lean sarcopenic	1.12	0.95–1.32	0.169
	Steatotic-dominant	0.93	0.73–1.18	0.545
Cerebrovascular disease	Balanced	Reference		
	Lean sarcopenic	1.14	0.85–1.52	0.384
	Steatotic-dominant	0.98	0.64–1.51	0.937

PDFF, MFI, and FFMV were standardized separately within men and women before k-means clustering (k=3). The resulting clusters comprised 4,573 Balanced, 4,545 Lean-sarcopenic, and 1,372 Steatotic-dominant participants. The proportions of men were 64.9%, 64.2%, and 64.1%, respectively. Cox models were adjusted for age, sex, BMI, diabetes, dyslipidemia, hypertension, smoking, ALT, AST, GGT, total bilirubin, albumin, and platelet count. HR, hazard ratio; CI, confidence interval; MASLD, metabolic dysfunction-associated steatotic liver disease; PDFF, proton density fat fraction; MFI, muscle fat infiltration; FFMV, fat-free muscle volume.

Supplementary Table S9. Associations of abdominal and muscle composition parameters with clinical outcomes in the MASLD cohort with FIB-4 < 1.45

Overall survival												
Variable	Unadjusted			Model 1			Model 2			Model 3		
	HR	95% CI	*P* value	HR	95% CI	*P* value	HR	95% CI	*P* value	HR	95% CI	*P* value
Liver PDFF (%)	0.99	0.95, 1.01	0.147	0.99	0.96, 1.02	0.530	0.99	0.96, 1.02	0.352	0.98	0.96, 1.02	0.452
VAT (L)	1.16	1.07, 1.26	< 0.001	1.05	0.96, 1.15	0.326	1.03	0.94, 1.13	0.579	1.03	0.94, 1.13	0.539
ASAT (L)	0.90	0.85, 0.94	< 0.001	0.98	0.92, 1.05	0.579	0.96	0.89, 1.04	0.366	0.96	0.89, 1.04	0.364
MFI (%)	1.19	1.12, 1.27	< 0.001	1.14	1.06, 1.22	< 0.001	1.11	1.03, 1.19	0.005	1.10	1.03, 1.19	0.007
Total thigh FFMV (L)	0.94	0.87, 1.01	0.083	0.98	0.89, 1.09	0.743	0.98	0.88, 1.09	0.718	0.98	0.88, 1.09	0.708
**Incidence of cardiovascular disease**												
Variable	Unadjusted			Model 1			Model 2			Model 3		
	HR	95% CI	*P* value	HR	95% CI	*P* value	HR	95% CI	*P* value	HR	95% CI	*P* value
Liver PDFF (%)	0.98	0.97, 1.00	0.047	0.99	0.97, 1.01	0.317	0.98	0.97, 1.00	0.073	0.99	0.97, 1.01	0.217
VAT (L)	1.22	1.16, 1.28	< 0.001	1.11	1.05, 1.18	< 0.001	1.05	0.99, 1.11	0.132	1.05	0.99, 1.11	0.118
ASAT (L)	0.91	0.88, 0.94	< 0.001	0.99	0.95, 1.03	0.722	1.00	0.96, 1.06	0.851	1.00	0.95, 1.05	0.695
MFI (%)	1.05	1.00, 1.10	0.072	1.01	0.96, 1.07	0.743	0.99	0.94, 1.05	0.693	0.98	0.93, 1.04	0.489
Total thigh FFMV (L)	0.93	0.88, 0.97	0.002	0.90	0.85, 0.96	0.002	0.93	0.87, 0.99	0.024	0.93	0.87, 0.99	0.020
**Incidence of cerebrovascular disease**												
Variable	Unadjusted			Model 1			Model 2			Model 3		
	HR	95% CI	*P* value	HR	95% CI	*P* value	HR	95% CI	*P* value	HR	95% CI	*P* value
Liver PDFF (%)	0.98	0.95, 1.01	0.159	0.99	0.96, 1.02	0.463	0.98	0.95, 1.01	0.215	0.98	0.94, 1.01	0.180
VAT (L)	1.23	1.12, 1.34	< 0.001	1.12	1.01, 1.24	0.031	1.08	0.97, 1.20	0.138	1.08	0.97, 1.20	0.140
ASAT (L)	0.87	0.82, 0.92	< 0.001	0.95	0.88, 1.03	0.197	0.98	0.89, 1.07	0.653	0.98	0.89, 1.07	0.598
MFI (%)	1.15	1.07, 1.24	< 0.001	1.09	1.00, 1.18	0.058	1.07	0.98, 1.17	0.115	1.08	0.99, 1.18	0.102
Total thigh FFMV (L)	0.91	0.83, 0.99	0.023	0.95	0.85, 1.07	0.425	0.98	0.87, 1.11	0.793	0.98	0.87, 1.10	0.759

Model 1: adjusted for age and sex.
Model 2: additionally adjusted for metabolic risk factors (BMI, diabetes, dyslipidemia, hypertension, and smoking).
Model 3: additionally adjusted for liver biochemical parameters (ALT, AST, GGT, total bilirubin, albumin, and platelet count).
HR, hazard ratio; CI, confidence interval; PDFF, proton density fat fraction; VAT, visceral adipose tissue; ASAT, abdominal subcutaneous adipose tissue; MFI, muscle fat infiltration; FFMV, fat-free muscle volume.

Supplementary Table S10. Fine–Gray competing-risk analysis of MRI body-composition parameters and incident cardiovascular and cerebrovascular disease in the MASLD cohort

Outcome	Variable	Adjusted sHR	95% CI	Adjusted *P* value
Cardiovascular disease	**Liver PDFF (%)**	0.98	0.97–1.00	0.028
	**VAT (L)**	1.06	1.01–1.11	0.021
	**ASAT (L)**	0.99	0.95–1.03	0.696
	**MFI (%)**	0.99	0.95–1.04	0.814
	**Total thigh FFMV (L)**	0.93	0.88–0.98	0.004
Cerebrovascular disease	**Liver PDFF (%)**	0.99	0.96–1.02	0.459
	**VAT (L)**	1.03	0.94–1.12	0.578
	**ASAT (L)**	0.95	0.87–1.04	0.271
	**MFI (%)**	1.09	1.01–1.17	0.026
	**Total thigh FFMV (L)**	0.96	0.87–1.06	0.432

Death before the respective cardiovascular or cerebrovascular event was treated as a competing event. Fine–Gray subdistribution hazard models were mutually adjusted for liver PDFF, VAT, ASAT, MFI, and total thigh FFMV, and further adjusted for age, sex, BMI, diabetes, dyslipidemia, hypertension, smoking, ALT, AST, GGT, total bilirubin, albumin, and platelet count. sHR, subdistribution hazard ratio; CI, confidence interval; MRI, magnetic resonance imaging; MASLD, metabolic dysfunction-associated steatotic liver disease; PDFF, proton density fat fraction; VAT, visceral adipose tissue; ASAT, abdominal subcutaneous adipose tissue; MFI, muscle fat infiltration; FFMV, fat-free muscle volume.
